# Anti-Xanthine Oxidase 5′-Hydroxyhericenes A–D from the Edible Mushroom *Hericium erinaceus* and Structure Revision of 3-[2,3-Dihydroxy-4-(hydroxymethyl)tetrahydrofuran-1-yl]-pyridine-4,5-diol

**DOI:** 10.1021/acsomega.3c07792

**Published:** 2023-11-21

**Authors:** Tawatchai Thongkongkaew, Narumol Jariyasopit, Sakda Khoomrung, Siraprapa Siritutsoontorn, Sarawut Jitrapakdee, Prasat Kittakoop, Somsak Ruchirawat

**Affiliations:** † Chemical Sciences Program, Chulabhorn Graduate Institute, Chulabhorn Royal Academy, Laksi, Bangkok 10210, Thailand; ‡ Siriraj Center of Research Excellence in Metabolomics and System Biology (SiCORE-MSB), Faculty of Medicine Siriraj Hospital, Mahidol University, Bangkok 10700, Thailand; § Siriraj Metabolomics and Phenomics Center, Faculty of Medicine Siriraj Hospital, Mahidol University, Bangkok 10700, Thailand; ∥ Department of Biochemistry, Faculty of Medicine Siriraj Hospital, Mahidol University, Bangkok 10700, Thailand; ⊥ Department of Biochemistry, Faculty of Science, Mahidol University, Bangkok 10400, Thailand; # Chulabhorn Research Institute, Kamphaeng Phet 6 Road, Laksi, Bangkok 10210, Thailand; ∇ Center of Excellence on Environmental Health and Toxicology (EHT), OPS, Ministry of Higher Education, Science, Research and Innovation, Bangkok 10400, Thailand

## Abstract

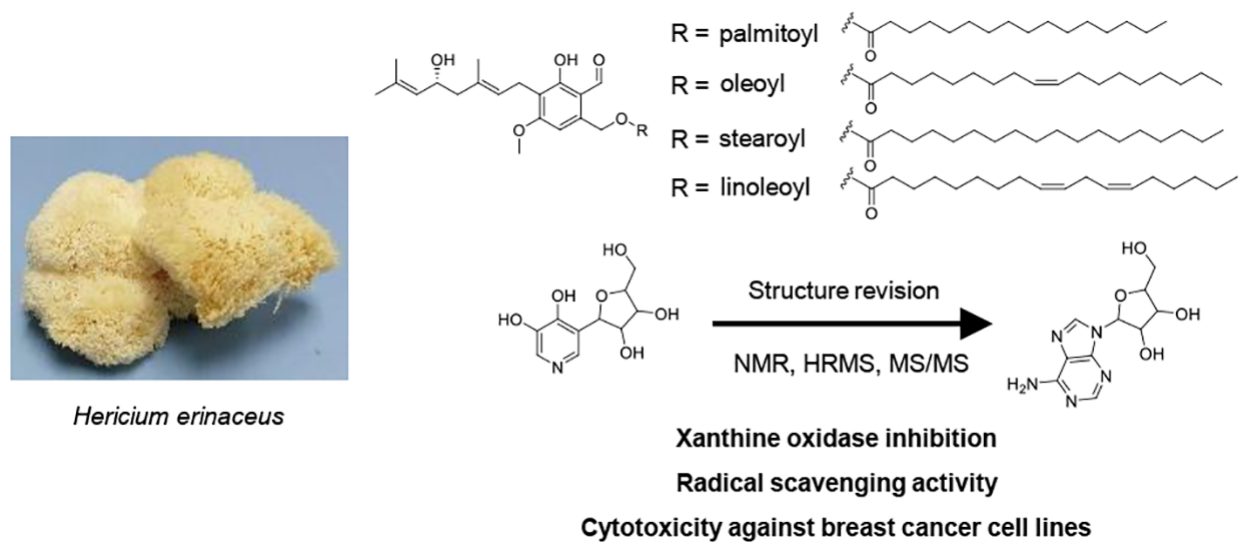

*Hericium erinaceus* is an edible mushroom with diverse pharmaceutical applications. Although this mushroom is an attractive source of natural products for cancer treatment, little is known about the bioactive compounds from this mushroom, which may possess antibreast cancer activity. Here, we report the isolation and structure elucidation of new compounds, 5′-hydroxyhericenes A–D (**1–4**) as an inseparable mixture, together with known compounds (**5–16**) from the fruiting body of *H. erinaceus*. Based on NMR spectroscopic data and MS fragmentation analysis, the structure of a previously reported natural product, 3-[2,3-dihydroxy-4-(hydroxymethyl)tetrahydrofuran-1-yl]-pyridine-4,5-diol (**5**), should be revised to adenosine (**6**). Compounds **1–4** inhibit xanthine oxidase activity, while compounds **6**, **9**, and **10** scavenge reactive oxygen species generated by xanthine oxidase. Moreover, hericerin (**13**) exhibits strong growth inhibitory activity against T47D breast cancer cells and, to a lesser extent, against MDA-MB-231 breast cancer and MRC-5 normal embryonic cells. Exposure of T47D and MDA-MB-231 cells slightly increased PARP cleavage, suggesting that the growth inhibitory effect of hericerin may be mediated through nonapoptotic pathways. Our results suggest that the bioactive compounds of mushroom *H. erinaceus* hold promise as antibreast cancer agents.

## Introduction

The edible mushroom *Hericium erinaceus* is native to North America, Europe, and Asia. The mushroom is considered a saprophyte because it usually grows on dead trees or dead parts of the living trees. Due to its large fruiting body and hanging spines, this mushroom is recognized by other names such as Lion’s mane, monkey head, and bearded Hedgehog mushroom. *H. erinaceus* attracts the attention of the scientific community due to the health-promoting effects of macromolecules and small molecules.^1,2^ Polysaccharides are the major macromolecules from the mushroom that possess anticancer, antioxidant, antihyperglycemic, and immunomodulating activities.^3^ Geranyl-resorcinols and cyathane terpenoids are two major types of small molecules from the fruiting body and mycelium, respectively.^4^ Hericenones and erinacines from *H. erinaceus* stimulate nerve growth factor synthesis, which mitigates the effect of neuronal cell death during aging and in age-related diseases, such as Alzheimer’s disease.^5−7^ Moreover, hericenone and erinacine derivatives exhibit α-glucosidase inhibition,^8^ antiosteoporotic activities,^9^ and cytotoxicity against various cancer cell lines.^10^

Cancer remains the leading cause of death worldwide, and breast cancer is the major concern for women, causing 684,996 deaths in 2020.^11^ Recently, the positive effect of xanthine oxidase on breast cancer has been described. This enzyme converts hypoxanthine to uric acid and generates reactive oxygen species. A high level of uric acid in the blood leads to the development of gout, which increases the risk of breast cancer.^12,13^ Although several anticancer drugs are available, the number of cancer patients still increases, partly due to the ability of cancer to develop resistance through various mechanisms, such as drug inactivation, drug efflux, and inhibition of cell death.^14^ In addition, anticancer drugs are expensive, limiting their accessibility to patients and their potential for adverse side effects. Thus, the search for effective, inexpensive, and safe anticancer drugs is highly demanding. Since edible mushrooms are commercially available and safe for humans (being used as food), they are attractive sources of new medicines in anticancer therapies.^15^

As part of our drug discovery research against cancer, we screened the crude extracts of edible mushrooms for anticancer agents. Crude extracts from *H. erinaceus* showed the most promising results, but there is little information available for breast cancer therapies using this mushroom. In this paper, we describe the isolation and structure elucidation of new geranyl-resorcinol derivatives and propose a structure revision of an alkaloid, 3-[2,3-dihydroxy-4-(hydroxymethyl)tetrahydrofuran-1-yl]-pyridine-4,5-diol, previously isolated from the mealworm *Tenebrio molitor*,^16^ and later from the leaves of *Pachyrhizus erosus*(17) and *Piper sarmentosum*,^18^ and the whole plants of *Houttuynia cordata* Thunb.^19^ Biological screening revealed potential drug candidates for breast cancer treatments. This information will be useful for the future development of anticancer drugs based on edible mushrooms.

## Results and Discussion

**Figure 1:**
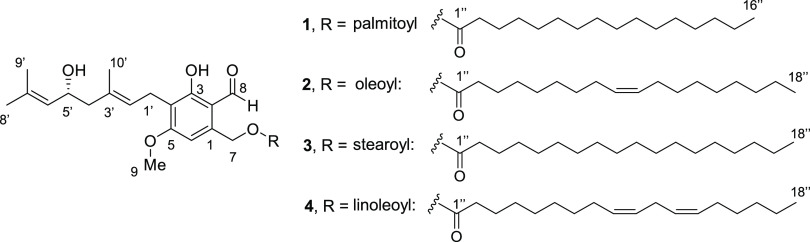
Structure of 5′-hydroxyhericenes A–D (**1–4**).

The hexane, ethyl acetate, and *n*-butanol extracts from the fruiting body of *H. erinaceus* were purified by sephadex-LH20, silica gel column chromatography, preparative thin layer chromatography, and HPLC to yield new compounds named 5′-hydroxyhericenes A–D (**1–4**) (Figure 1). Known compounds were also isolated as follows: 3-[2,3-dihydroxy-4-(hydroxymethyl) tetrahydrofuran-1-yl]-pyridine-4,5-diol (**5**)^16^ (it is revised to adenosine (**6**) in this study), hericenes A and C (**7** and **8**),^20^ hericenones C and D (**9** and **10**),^6^ erinacerin B (**11**),^21^ hericenone A (**12**),^22^ hericerin (**13**),^23−25^ herierin III (**14**),^26^ herierin IV (**15**),^27^ erinapyrone C (**16**),^20^ nicotinamide, and linoleic acid. For known compounds, their structures were established by analysis of 1D and 2D NMR, and HRMS data, as well as by comparison of the NMR spectroscopic data with literature. The structures of new compounds were elucidated by analysis of NMR, HRMS, and GC-MS data, and their absolute configuration was established by Mosher’s method.

**Figure 2:**
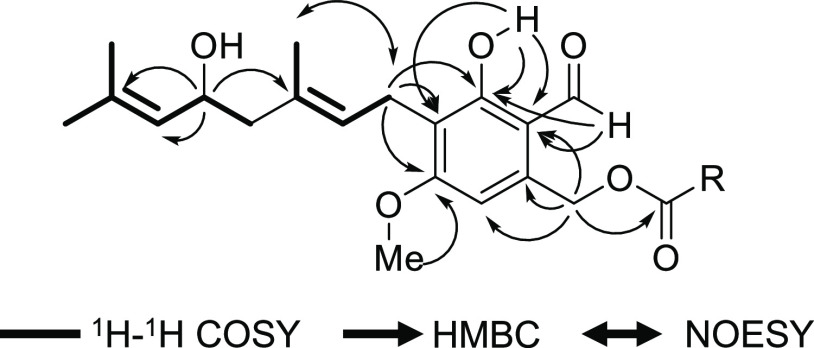
Key ^1^H–^1^H COSY, HMBC, and NOESY correlations for the 5′-hydroxygeranyl-resorcinol in compounds **1–4**. R represents the fatty acid.

**Figure 3:**
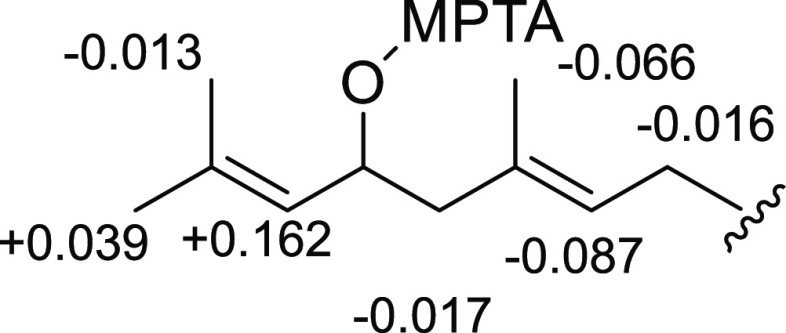
Values of Δδ_*S*-*R*_ of the (*R*) and (*S*)-MTPA esters of the 5′-hydroxygeranyl moiety in compounds **1–4**.

Compounds **1–4** were obtained as an inseparable mixture and were isolated as a pale yellow solid. Their molecular formulas were determined as C_35_H_56_O_6_, C_37_H_60_O_6_, C_37_H_58_O_6_, and C_37_H_56_O_6_ based on the following ESI-HRMS data: *m*/*z* 595.3965 [M + Na]^+^, calculated 595.3969; *m*/*z* 623.4276 [M + Na]^+^, calculated 623.4282; *m*/*z* 621.4130 [M + Na]^+^, calculated 621.4126; and *m*/*z* 619.3974 [M + Na]^+^, calculated 619.3969. The IR spectrum showed absorptions at 3532, 1737, and 1622 cm^–1^ indicating the presence of hydroxyl, carbonyl, and aryl-alkene functional groups. Compounds **1–4** contain two parts: fatty acids and geranyl-resorcinols. The fatty acids were identified by GC-MS, and the geranyl-resorcinols were identified by NMR. Methanolysis of the mixture of **1–4** afforded methyl esters which were subjected to GC-MS. The retention times and the mass spectra of fatty acid methyl esters from the hydrolysate were compared with those obtained from a 37 FAME mix standard and the National Institute of Standards and Technology (NIST) mass spectral library (Figures S13 and S14). The fatty acids in a hydrolysate were identified as palmitic, stearic, oleic, and linoleic in an approximately 21:11:2:1 ratio. According to the GC-MS data, **1** and **3** are major compounds, while **2** and **4** are present in minute quantities. To isolate compounds **1–4** in pure forms, we first used a reversed phase HPLC and conditions, described for purification of other hericene derivatives.^4,6^ However, a semipurified mixture of compounds **1–4** precipitated under those reported conditions. Thus, we then used normal phase HPLC to purify a mixture of compounds **1–4**, however, these closely related compounds could not be separated by a normal phase HPLC. Since attempts to separate compounds **1–4** by normal phase HPLC had met with failure, the structure of the geranyl-resorcinol parts was elucidated by NMR using an inseparable mixture of **1–4**. It is worth mentioning that the geranyl-resorcinol moiety in compounds **1**–**4** had similar ^1^H and ^13^C resonances, showing only one set of signals (Figures S1 and S2). ^1^H and ^13^C NMR (Table 1) showed signals of chelated hydroxyl (δ_H_ 12.39), one aldehyde (δ_H_ 10.10), three aromatic/olefinic methine (δ_H_ 6.52, 5.30, and 5.11), four methyl (δ_H_ 3.91, 1.81, 1.68, and 1.66), three methylene (δ_H_ 5.31, 3.37, and 2.11), and one methine (δ_H_ 4.40) groups. ^1^H–^1^H COSY correlations revealed the presence of 5′-hydroxygeranyl (Figure 2). The hydroxyl group was located at C5′ of geranyl based on HMBC correlations from H5′ (δ_H_ 4.40) to C7′ (δ_C_ 134.7), C6′ (δ_C_ 127.4), and C3′ (δ_C_ 132.1) Moreover, the configuration of the double bond at C2′ was established as *E* based on NOESY correlation between H1′ (δ_H_ 3.37) and H10′ (δ_H_ 1.81) (Figure 2). The ^1^H and ^13^C NMR data of the 5′-hydroxygeranyl unit in compounds **1–4** are similar to those of the 5′-hydroxygeranyl in erinacerin B.^21^ Next, HMBC correlations from H1′ (δ_H_ 3.37) to C5 (δ_C_ 163.4), C4 (δ_C_ 117.3), and C3 (δ_C_ 162.8) connected the 5′-hydroxygeranyl to the resorcinol ring at C4 (Figure 2). Moreover, HMBC correlations from H8 (δ_H_ 10.10) to C3 (δ_C_ 162.8) and C2 (δ_C_ 112.9) and from H9 (δ_H_ 3.91) to C5 (δ_C_ 163.4) located the aldehyde and methoxy groups at C2 and C5 of the resorcinol ring, respectively. The fatty acid was connected to C1 based on the HMBC correlations from H7 (δ_H_ 5.31) to C1″ (δ_C_ 173.2), C1 (δ_C_ 138.7), C2 (δ_C_ 112.9), and C6 (δ_C_ 105.5) (Figure 2). To determine the absolute configuration of the hydroxyl group in the 5′-hydroxygeranyl moiety, we employed a modified Mosher’s method. According to the values of Δδ_*S*-*R*_ of the (*S*) and (*R*)-MTPA esters, the absolute configuration at C5′ was concluded to be *R*. (Figure 3)

**Table 1:** ^1^H and ^13^C Data of 5′-Hydroxygeranyl-Resorcinol Part of Compounds **1–4** in CDCl_3_

**position**	**δ**_**C**_, **type**^a^	**δ**_**H**_, **multiplicity (*****J*****in Hz)**^b^
1	138.7, C	
2	112.9, C	
3	162.8, C	
3-OH		12.39, s
4	117.3, C	
5	163.4, C	
6	105.5, CH	6.52, s
7	62.8, CH_2_	5.31, s
8	193.1, CHO	10.10, s
9	55.9, CH_3_	3.91, s
1′	21.4, CH_2_	3.37, m
2′	125.9, CH	5.30, m
3′	132.1, C	
4′	48.2, CH_2_	2.11, m
5′	65.6, CH	4.40, ddd (8.5, 8.5, 4.7)
6′	127.4, CH	5.11, dt (8.4, 1.3)
7′	134.7, C	
8′^c^	18.1, CH_3_	1.66, d (1.2)
9′^c^	25.7, CH_3_	1.68, d (1.1)
10′	16.2, CH_3_	1.81, brs
1″	173.2, C	
2″	34.2, CH_2_	2.33, t (7.5)
3″	24.8, CH_2_	1.70–1.55, m
16″ or 18″	14.1, CH_3_	0.87, t (6.7)

a ^13^C NMR chemical shifts of the 5′-hydroxygeranyl-resorcinol unit in compound**s 1–4** are identical. The chemical shifts at 34.2, 31.9, 29.7–29.1, 24.8, and 22.7 ppm are methylene carbons of fatty acids.

b ^1^H chemical shifts of the 5′-hydroxygeranyl-resorcinol unit in compound**s 1–4** are identical. The olefinic protons in oleic part **2** and in linoleic part **4** could not be identified due to overlapped signals at 5.3 ppm. The methylene protons at C11″ of the linoleic acid part in **4** have similar signal intensity to proton satellite peaks.

c Interchangeable signals.

**Figure 4:**
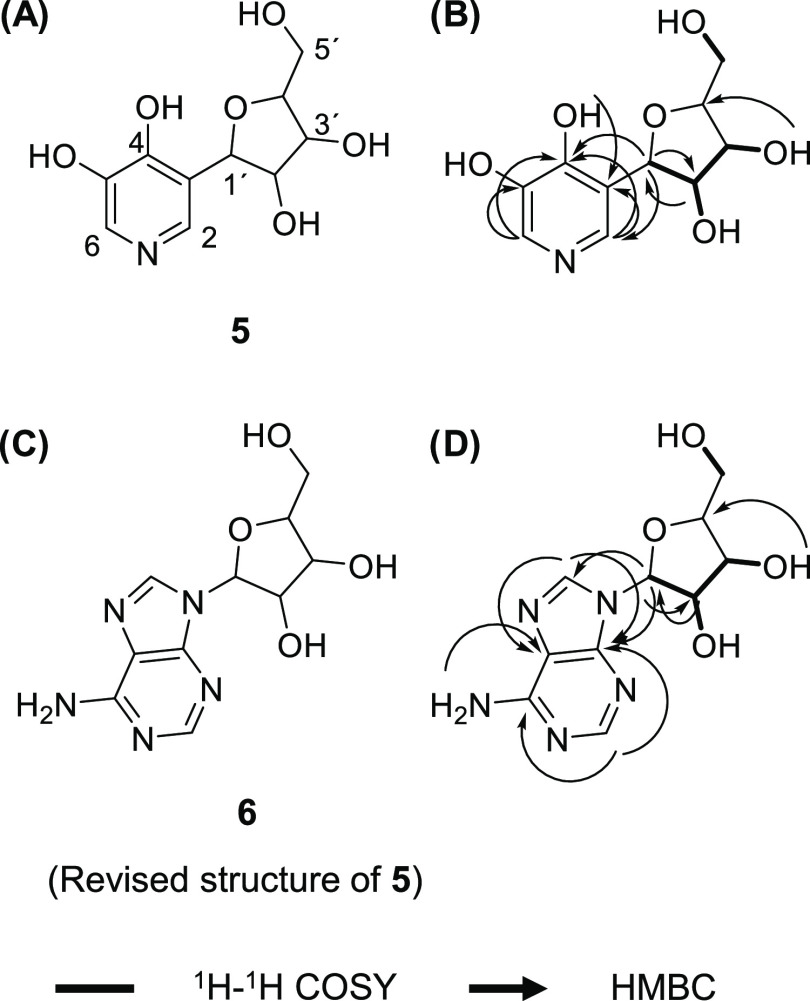
(A) Structure of 3-[2,3-dihydroxy-4-(hydroxylmethyl)tetrahydrofuran-1-yl]-pyridine-4,5-diol (**5**) was isolated from the mealworm *T. molitor*. (B) Key ^1^H–^1^H COSY and HMBC correlations of the initial proposed structure of compound **5** in this study. (C) The structure of compound **5** should be revised to adenosine (**6**) (D) Key ^1^H–^1^H COSY and HMBC correlations of adenosine (**6**) in this study.

During the purification of bioactive compounds from the *n*-BuOH extract of *H. erinaceus*, we obtained compound **5**, and analysis of NMR data revealed that a structure of **5** might be 3-[2,3-dihydroxy-4-(hydroxyl methyl)tetrahydrofuran-1-yl]-pyridine-4,5-diol (**5**) (Figure 4A,4B). This alkaloid was first isolated from the mealworm *T. molitor*,^16^ and later from the leaves of *P. erosus*(17) and *P. sarmentosum*,^18^ and the whole plants of *H. cordata* Thunb.^19^ Compound **5** had identical ^1^H and ^13^C NMR data (DMSO-*d*_6_) to those of the alkaloid from the mealworm (Table 2) as well as the published NMR data of the alkaloid from plants.^17,19^ However, the ESI-HRMS data of compound **5** showed the *m*/*z* at 268.1039 [M + H]^+^, calculated at 268.1040, corresponding to the molecular formula C_10_H_13_O_4_N_5_, which did not match with the structure of the alkaloid from the mealworm. This raised the possibility that the previously proposed structure for **5** might be incorrect. Structure elucidation of natural products relies mainly on the analysis of NMR spectroscopic data, but a large number of misassigned structures of natural products in literature remains exceedingly high.^28,29^ Combined information from various techniques is essential for firmly establishing the structure of both known and unknown natural products. Reinvestigation of the NMR data suggested that compound **5** might be adenosine (**6**), based on the analysis of ^1^H–^1^H COSY and HMBC correlations (Figure 4C,4D). To further confirm the structure of compound **5**, an MS/MS fragmentation study was performed and the results were compared with a commercially available adenosine. The fragmentation patterns of compound **5** and the commercial compound are similar, with the *m*/*z* at 136.0619 [M + H]^+^, calculated 136.0618, corresponding to adenine (Figure S15).^30^ Finally, we found that the ^1^H and ^13^C NMR spectra of the isolated compound were identical to those of the standard adenosine (Figures S16 and S17), conclusively confirming that compound **5** was an adenosine (**6**). Therefore, we proposed that the structure of 3-[2,3-dihydroxy-4-(hydroxylmethyl)tetrahydrofuran-1-yl]-pyridine-4,5-diol (**5**) should be revised to adenosine (**6**). Moreover, 3-[2,3-dihydroxy-4-(hydroxylmethyl)tetrahydrofuran-1-yl]-pyridine-4,5-diol (**5**) was previously reported to exhibit antiinflamation^31^ and antiwrinkle activities.^32^ Coincidently, both biological activities were previously described for adenosine (**6**).^33,34^

**Table 2:** ^1^H and ^13^C Data of Compound **5** in DMSO-*d*_6_

	**this study**		**from patent**(16)	
**position**	**δ**_**C**_, **type**^a^	**δ_H_**, **multiplicity(*J* in Hz)**^a^	**δ**_**C**_, **type**^b^	**δ_H_**, **multiplicity(*J* in Hz)**^b^
2	139.9, CH	8.34, s	140.0, CH	8.35, s
3	119.3, C		119.4, C	
4	149.0, C		149.1, C	
5	156.1, C		156.2, C	
6	152.4, CH	8.13, s	152.4, CH	8.14, s
1′	87.8, CH	5.87, d (6.2)	87.9, CH	5.87, d (5.7)
2′	73.4, CH	4.61, ddd (5.6, 5.6, 5.6)	73.5, CH	4.61, brs
3′	70.6, CH	4.14, ddd (3.7, 3.7, 3.4)	70.7, CH	4.14, brs
4′	85.8, CH	3.96, ddd (3.3, 3.3, 3.3)	85.9, CH	3.96, brs
5′	61.6, CH_2_	3.67, ddd (11.9, 4.0, 3.8)	61.7, CH_2_	3.69–3.66, m
		3.55, m		3.57–3.55, m
4-OH		7.33, brs		7.34, s
5-OH		7.33, brs		7.34, s
2′–OH		5.43, m		5.45, s
3′–OH		5.20, brd (4.2)		5.19, s
5′–OH		5.43, m		5.45, s

a Measured in 75 MHz for carbon and 300 MHz for proton.

b Measured in 125 MHz for carbon and 500 MHz for proton.

It is urgent to report a structure revision of 3-[2,3-dihydroxy-4-(hydroxymethyl)tetrahydrofuran-1-yl]-pyridine-4,5-diol to prevent the spread of data about this misassigned structure of a natural product. For example, a recent article proposed that compound **5** (misassigned structure) could be used as a chemotaxonomic marker to distinguish *H. cordata* from other plants.^19^ This has become a serious issue if using the misassigned structure of a natural product as a chemotaxonomic marker. Upon the example given above, the misassigned natural product, compound **5**, should be revised to adenosine (**6**) as soon as possible.

Compounds **1**–**4** and **6**–**16** were evaluated for their inhibitory activity against xanthine oxidase (IXO), diphenyl picrylhydracyl radical (DPPH), and reactive oxygen species generated by xanthine oxidase (XXO), as well as their cytotoxicity against T47D and MDA-MB-231 breast cancer cells and MRC-5 normal cell lines (Table S1).

Only an inseparable mixture of 5′-hydroxyhericenes A–D (**1–4**) showed inhibitory activity against IXO with an IC_50_ value of 7.3 ± 0.6 μg mL^–1^. Comparing results from compounds **1–4** and hericene derivatives **7–10**, the free hydroxyl group at C5′ of the geranyl-resorcinol part seems to be important for IXO activity; however, the role of fatty acids cannot be ruled out. For antioxidant activity, tested compounds show weak inhibitory activity against DPPH, while an inseparable mixture of hericenones C and D (**9** and **10**) and adenosine (**6**) inhibit XXO with IC_50_ values of 60.8 ± 6.1 μg mL^–1^and 133.0 ± 6.8 μg mL^–1^ (498.0 ± 25.5 μM), respectively. Despite its negligible activity in antioxidant assays, adenosine is known to prevent cellular damage from reactive oxygen species by activating cellular antioxidant enzymes.^35^ In addition, the standardized aqueous extract of *H. erinaceus*, which contains adenosine as a major component, reduces intracellular reactive oxygen species from corticosterone-induced oxidative stress in PC-12 cells, a model mimicking depression.^26^

**Figure 5:**
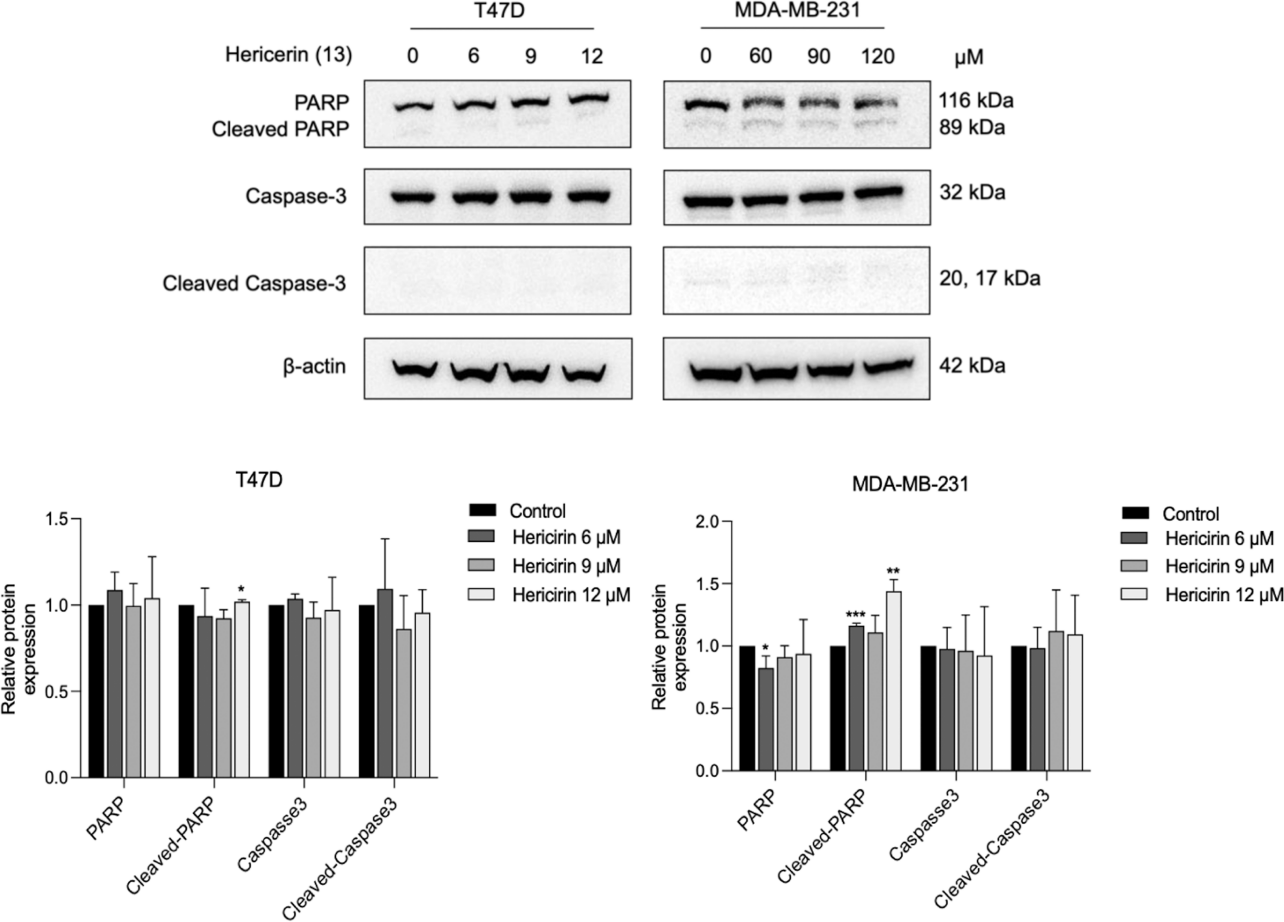
Effect of hericerin (**13**) on the apoptosis marker. T47D and MDA-MB-231 cells were treated with various concentrations of Hericerin for 48 h prior to detection of full length PARP (116 kDa), cleaved PARP (89 kDa), full length Caspase-3 (32 kDa), and cleaved Caspase 3 (20 and 17 kDa) by Western blot analysis (Upper panel) (*n* = 3). The relative abundance of full length PARP, cleaved PARP, full length Caspase-3, and cleaved Caspase-3 in T47D and MDA-MB-231 cell lines are shown in the lower panel, respectively. *p < 0.05, **p < 0.01; ***p < 0.001.

For cytotoxicity, tested compounds exhibited weak activity, except hericerin (**13)** that suppresses the growth of T47D and MDA-MB-231 cancer cell lines with IC_50_ values of 5.9 ± 1.6 and 57.5 ± 5.4 μM, respectively. In a further assay with the MRC-5 normal cell line, compound **13** showed growth inhibitory activity with an IC_50_ value of 79.6 ± 13.7 μM. Since the IC_50_ values of hericerin against MDA-MB-231 and MRC-5 are higher than 10 μM, hericerin is considered a noncytotoxic agent for those cell lines. The selectivity index between the T47D cancer cell line and the MRC-5 normal cell line is 13.5. In the present work, hericerin (**13**) shows strong cytotoxic activity against the T47D cell line but not against MDA-MB-231 cells. In addition, this compound was previously reported to be noncytotoxic to MCF-7 cancer cell line.^36^ This means that the growth inhibitory effect of hericerin depends on the types of breast cancer cell. T47D, MDA-MB-231, and MCF-7 are popular models in breast cancer studies. The T47D and MCF-7 are hormone-dependent breast cancer cells, but MDA-MB-231 is a hormone-independent breast cancer cell. T47D is susceptible to progesterone, whereas MCF-7 does not respond to progesterone in the presence of estrogen.^37^ Thus, hericerin (**13**) may be a good candidate to develop an anticancer agent against progesterone-sensitive breast cancer cells. Moreover, hericerin was reported to inhibit the growth of A549, SK-OV-3, SK-MEL-2, HCT-15, and HL-60 cancer cell lines.^10,38^ To determine the possible mechanism, we performed an apoptosis assay. First, T47D and MDA-MB-231 breast cancer cells were exposed to compound **13** for 48 h, and the abundance of two apoptotic markers, namely, PARP and procaspase 3, was determined by Western blot. As shown in Figure 5, compound **13** slightly induced PARP cleavage in both T47D and MDA-MB-231 cells, while it had no effect on Caspase-3 cleavage. This result suggests that the inhibitory effect of hericerin (**13**) may not be entirely attributed to the apoptosis pathway in both breast cancer cell lines.

Breast cancer is a complex disease. The cancer is grouped into at least five subtypes, and each subtype has a different prognosis and response to treatments.^39^ In the advanced stages, the treatment is challenging because breast cancer cells can detach from the primary tumor site and spread to the bone, lung, and liver through a process called metastasis. It was reported that febuxostat, a xanthine inhibitor, suppresses breast cancer metastasis *in vitro* and reduces tumor size in a mouse model.^40^ Moreover, this inhibitor is used to treat gout, one of the risk factors for breast cancer. Our results suggest that *H. erinaceus* is a potential candidate to develop medicines for breast cancer therapies as the fruiting body contains a cocktail of compounds with cytotoxic and xanthine oxidase inhibitory activities. The combined effect of bioactive compounds can not only inhibit the growth of breast cancer cells, but also help reduce the level of uric acid in the blood, and possibly the metastasis that will complicate breast cancer therapies.

In summary, we reported the isolation and structure elucidation of 5′-hydroxyhericenes A–D (**1–4**) as an inseparable mixture. In addition, the structure of 3-[2,3-dihydroxy-4-(hydroxylmethyl)tetrahydrofuran-1-yl]-pyridine-4,5-diol (**5**), previously isolated from the mealworm and plants, should be revised to adenosine (**6**). Compounds **1–4** inhibit xanthine oxidase, while **6** and **9**, **10** selectively inhibit reactive oxygen species generated by this enzyme. Hericerin (**13**) shows strong cytotoxicity against T47D breast cancer cells, while this compound weakly inhibits the growth of MDA-MB-231 breast cancer and MRC-5 normal cell lines. The mechanism of inhibition of **13** possibly occurs via nonapoptotic pathway.

## Experimental Section

### General Experimental Procedures

^1^H and ^13^C NMR spectroscopic data were recorded on a Bruker Avance III HD 300 MHz. The solvent residue peaks of CDCl_3_ (δ_H_ = 7.26 ppm, δ_C_ = 77.0 ppm) and DMSO-*d*_6_ (δ_H_ = 2.50 ppm, δ_C_ = 39.5 ppm) were used as references. IR spectrum was recorded on a Thermo Scientific, Nicolet iS 5, ATR. ESI-HRMS and MS/MS spectra were recorded on a Thermo Scientific orbitrap Q Exactive Focus. GC-MS analysis was performed on a GC coupled with a time-of-flight MS (LECO Pegasus BT, Leco Corporation, St. Joseph, MI) using a ZB-FAME column (30 m × 0.25 mm × 0.2 μm, Zebron). Column chromatography was performed on a Sephadex LH20 and Merck silica gel 60. Preparative thin layer chromatography (PTLC) was performed on Merck silica gel 60 PF_254_. HPLC was carried out on an Agilent Tehnologies 1200 series with UV detector using a Hichrom C18 reversed phase (5 μm, 250 × 4.6 mm, flow rate 1 mL min^–1^) column, an Agilent Tehnologies 1260 series with UV detector using a Phenomenex Luna C18 reversed phase (5 μm, 250 × 21.2 mm, flow rate 10 mL min^–1^), and a SpectraSystem P4000 using a Phenomenex Luna silica (2) (10 μm, 250 × 10.0 mm, flow rate 5 mL min^–1^). Technical grade solvents were distilled before use. HPLC- grade solvents were used for the HPLC analysis and purification. Adenosine was purchased from Sigma-Aldrich. A standard mix of 37 FAMEs was purchased from Restek (Bellefonte, PA).

### Fungal Materials

Fruiting bodies and mycelium of *H. erinaceus* were purchased from the Phothong mushroom farm, Nonthaburi Province, Thailand. The mycelium was grown on potato dextrose agar (PDA), 28 °C, for 7 days. The identity of the fungus was confirmed by comparing the nucleotide region of ITS rDNA with the GenBank sequence database. The NCBI accession number is OR327063. This fungal isolate was designated as CGI:F-002 and was deposited at the Chemical Science Program, Chulabhorn Graduate Institute, Thailand.

### Extraction and Isolation

Fruiting bodies (1 kg) of *H. erinaceus* CGI:F-002 were extracted with 5L of MeOH, three times, each for 2 days. The methanol layer was combined, concentrated, and extracted sequentially with hexane, EtOAc, and *n*-BuOH yielding crude extracts of 5.9, 1.5, and 3.4 g, respectively.

The hexane extract was purified by silica gel eluted with a 0–100% EtOAc:hexane gradient system to yield fractions F1–F5. Fraction F1 was purified by silica gel eluted with 5% EtOAc:hexane to yield an inseparable mixture of hericenes A and C (**7** and **8**, 377.5 mg). Fraction F2 was purified by silica gel eluted with 5% EtOAc:CH_2_Cl_2_ to yield fractions F6–F11. Fraction F7 contains an inseparable mixture of hericenones C and D (**9** and **10**, 676.1 mg). Fractions F9 and F10 were combined, purified by PTLC eluted with 20% EtOAc:hexane, and then with normal phase HPLC eluted with a 1–5% EtOH:hexane gradient system to yield 5′-hydroxyhericenes A–D (**1–4**) as an inseparable mixture (4.7 mg). Fraction F3 was purified by HPLC eluted with a 40–100% CH_3_CN:H_2_O (0.01% TFA) gradient system to yield fractions F12–F16. Fraction F16 contains linoleic acid (48.5 mg). Fraction F13 was purified by silica gel eluted with 10% EtOAc:CH_2_Cl_2_ to yield hericenone A (**12**, 8.9 mg). Fraction F15 was purified by silica gel eluted with a 20–60% EtOAc:hexane gradient system and then by PTLC eluted with 30% EtOAc:hexane to yield hericerin (**13**, 21.7 mg). Fraction F4 was purified by Sephadex LH20 eluted with MeOH and then by PTLC eluted with 10%EtOAc:CH_2_Cl_2_ to yield hericerin (**13**, 5.5 mg) and erinacerin B (**11**, 4.2 mg). Fraction F5 was purified by silica gel eluted with 0–50% MeOH:CH_2_Cl_2_ gradient system, by silica gel eluted with 100% EtOAc, and then by PTLC eluted with 20% EtOAc:hexane to yield hericenone C and D (**9** and **10**, 8.5 mg).

The EtOAc extract was purified by Sephadex LH20 eluted with MeOH to yield fractions F17-F23. Fractions F19 and F20 were combined and purified by silica gel eluted with a 0–100% MeOH:CH_2_Cl_2_ gradient system to yield fractions F24–F34. Fraction F25 was purified by silica gel eluted with 60% EtOAc:CH_2_Cl_2_ and then by PTLC eluted with 40% EtOAc:CH_2_Cl_2_ to yield hericerin (**13**, 4.7 mg). Fraction F28 was purified by silica gel eluted with 60% EtOAc:CH_2_Cl_2_ to yield erinapyrone C (**16**, 4.1 mg). Fraction F30 was purified by silica gel eluted with 5% EtOH:EtOAc to yield fractions F35–F40. Fraction F37 was purified by silica gel eluted with 5% MeOH:EtOAc to yield herierin IV (**15**, 8.6 mg). Fraction F40 was purified by silica gel eluted with 10% MeOH:CH_2_Cl_2_ to yield herierin III (**14**, 2.0 mg). Fraction F31 was purified with silica gel to yield herierin III (**14**, 36.5 mg). Fractions F22 and F23 were combined and purified by silica gel eluted with 20% MeOH:CH_2_Cl_2_ to yield adenosine (**6**, 4.0 mg).

The *n*-BuOH extract was purified by Sephadex LH20 eluted with MeOH to yield fractions F41–F46. Fraction F42 was purified by silica gel eluted with 20% MeOH:CH_2_Cl_2_ to yield fractions F47–F52. Fraction F49 was purified by silica gel eluted with 10% MeOH:EtOAc to yield fractions F53–F57. Fractions F55 and F56 were combined and purified by silica gel eluted with 10% MeOH:CH_2_Cl_2_ to yield herierin IV (**15**, 28.4 mg). Fraction F57 contained herierin III (**14**, 149.6 mg). Fraction F50 was purified by silica gel eluted with 20% MeOH:EtOAc to yield fractions F58–F61. Fractions F58–F60 were combined and purified by PTLC with 10% MeOH:CH_2_Cl_2_ to yield nicotinamide (2 mg) and herierin III (**14**, 6.9 mg). Fractions F43 and F44 were combined and purified by silica gel eluted with 20% MeOH:CH_2_Cl_2_ to yield adenosine (**6**, 21.8 mg).

#### 5′-Hydroxyhericenes A–D (**1–4**)

Pale yellow solid; IR (ATR) 3532, 1737, 1622 cm^–1^. ^1^H and ^13^C NMR data, see Table 1; ESI-HRMS *m*/*z* 595.3965 [M + Na]^+^, calculated 595.3969, Δ*m* −0.6 ppm; *m*/*z* 621.4130 [M + Na]^+^, calculated 621.4126, Δ*m* 0.7; *m*/*z* 623.4276 [M + Na]^+^, calculated 623.4282, Δ*m* −1.0 ppm; *m*/*z* 619.3974 [M + Na]^+^, calculated 619.3969, Δ*m* 0.8.

#### Adenosine (**6**) from *H. erinaceus*

Pale yellow solid; ^1^H and ^13^C NMR data, see Table 1; ESI-HRMS *m*/*z* 268.1039 [M + H]^+^, calculated 268.1040, Δ*m* −0.3.

### Preparation of the (*S*) and (*R*)-MTPA Esters of **1–4**

An inseparable mixture of **1–4** (1 mg) was dissolved in 200 μL of dried CH_2_Cl_2_. Next, **4-**dimethylaminopyridine (DMAP) (10 mg) and 10 μL of (*R*)-MTPA-Cl were added to a solution. The reaction was stirred at room temperature. After overnight, 200 μL of 0.1 M hydrochloric acid was added and the reaction mixture was extracted with EtOAc. After that, the organic layer was extracted again with 200 μL of 1 M sodium hydrogen carbonate. The water layer was discarded, while the organic layer was collected and evaporated to yield 0.3 mg of (*S*)-MTPA ester. The presence of the ester was monitored by TLC and its identity was confirmed by ESI-HRMS. A similar procedure was performed for (*R*)-MTPA ester. The chemical shift differences were assigned based on ^1^H NMR and ^1^H–^1^H-COSY experiments.

### Hydrolysis of Fatty Acid Side Chain and GC-MS Analysis

An inseparable mixture of **1–4** (0.5 mg) was dissolved in 250 μL of 0.1 M KOH in MeOH and then the reaction mixture was stirred in an ice bath. After overnight, the reaction was quenched by adding 250 μL of 0.1 M HCl. Next, the reaction mixture was extracted with hexane. The hexane layer was combined, evaporated, redissolved in 200 μL of analytical grade hexane, and analyzed by GC-MS. The front inlet was operated in the split mode (50:1) at 250 °C. The sample was separated on a ZB-FAME at the constant flow, using helium as a carrier gas with a total flow of 1 mL min^–1^. The GC oven temperature started at 40 °C (5 min hold) and then ramped to 238 °C at 3 °C min^–1^ (9 min hold). Temperatures at the transfer line and ion source were both 250 °C. The MS data were acquired at the acquisition rate of 10 spectra s^–1^ scanning from *m*/*z* 45–650. MS data process was performed using ChromaTOF software (version 5.32, Leco Corp.).

### Cytotoxicity Assays

Cytotoxicity assays were evaluated against the following cell lines: T-47D ATCC, HTB-133 (hormone-dependent breast cancer), and MRC-5 ATCC, CCL171 (normal embryonic lung) from the American Type Culture Collection (ATCC, Manassas, VA) as well as MDA-MB-231 ((hormone-independent breast cancer) from M.D. Anderson Cancer Center, Houston, TX). Doxorubicin hydrochloride was used as a positive control, and DMSO was used as a solvent and a negative control. The cutoff concentration is 50 μg mL^–1^. Cell viability was determined using the MTT assay. The IC_50_ value was calculated from the dose–response curve as the concentration that inhibits the cell growth by 50% in comparison with the negative control following 48 h of exposure of each tested compound (*n* = 4).

### Assays for Antioxidant, Radical Scavenging, and Xanthine Oxidase Inhibitory Activities

The assays were evaluated against DPPH (diphenyl picrylhydracyl radical), XXO (reactive oxygen species generated by xanthine oxidase), and IXO (inhibition of xanthine oxidase). DMSO was used as a solvent and a negative control. Ascorbic acid, gallic acid, and allopurinol were used as positive controls for the DPPH, XXO, and IXO assays, respectively. The cutoff concentration is 100 μg mL^–1^ for DPPH and 200 μg mL^–1^ for XXO and IXO. The IC_50_ value was calculated from the dose–response curve as the concentration that inhibits the reactive oxygen species or enzymatic activity by 50% in comparison with the negative control (*n* = 3).

### Apoptosis Assay

Detections of apoptotic markers, PARP and Caspase-3 were determined by Western blot analysis. Three ×10^5^ cells of MDA-MB-231 and T47D cell lines were plated into 6-well plates for 24 h before treating cells with various concentrations of hericerin for 48 h. Thirty μg of protein lysates were subjected to 7.5 or 12% SDS-polyacrylamide gel electrophoresis under reducing conditions. The proteins were transferred to PVDF membranes using a Semi-Dry Transfer Cell (Bio-Rad). The blots were incubated in a blocking buffer for 1 h before being incubated with anti-PARP (Cell signaling), anti-Caspase-3 (Santa Cruz), or anti-β-actin antibody (Sigma). Membranes were incubated with antirabbit or antimouse IgG secondary antibodies. The bands on membranes were detected by adding the chemiluminescent HPR substrate (Merck Millipore) and visualized using an enhanced chemiluminescence imaging system (Syngene).

## Supporting Information Available

Antioxidant, xanthine oxidase inhibition, and cytotoxicity of compounds **1–4** and **6–16**, 1D and 2D-NMR, ESI-HRMS, MS/MS of compounds **1–4**, GC-MS analysis of fatty acids, a comparison of ^1^H and ^13^C NMR between adenosine (**6**) isolated from *H. erinaceus* and a reference, and MS/MS fragmentation of adenosine (PDF)
