# Supplementary material for: Anti-Xanthine Oxidase 5′-Hydroxyhericenes A–D from the Edible Mushroom Hericium erinaceus and Structure Revision of 3-[2,3-Dihydroxy-4-(hydroxymethyl)tetrahydrofuran-1-yl]-pyridine-4,5-diol
Source: ACS Omega. 2023 Nov 21;8(48):46284–91. doi: 10.1021/acsomega.3c07792 (PMC10701869; doi:10.1021/acsomega.3c07792)
Supplement: Supplementary file 1 — ao3c07792_si_001.pdf [file ao3c07792_si_001.pdf]

SUPPORTING INFORMATION

Anti-Xanthine Oxidase 5´-Hydroxyhericenes A-D from the Edible Mushroom *Hericium erinaceus* and Structure Revision of 3-[2,3-Dihydroxy-4-(hydroxymethyl)tetrahydrofuran-1-yl]-pyridine-4,5-diol

Tawatchai Thongkongkaew[1*], Narumol Jariyasopit[2,3], Sakda Khoomrung[2,3,4], Siraprapa Siritutsoontorn[5], Sarawut Jitrapakdee[5], Prasat Kittakoop[1,6,7], Somsak Ruchirawat[1,6,7]

[1]Chemical Sciences Program, Chulabhorn Graduate Institute, Chulabhorn Royal Academy, Laksi, Bangkok 10210, Thailand

[2]Siriraj Center of Research Excellence in Metabolomics and System Biology (SiCORE-MSB), Faculty of Medicine Siriraj Hospital, Mahidol University, Bangkok 10700, Thailand

[3]Siriraj Metabolomics and Phenomics Center, Faculty of Medicine Siriraj Hospital, Mahidol University, Bangkok 10700, Thailand

[4]Department of Biochemistry, Faculty of Medicine Siriraj Hospital, Mahidol University, Bangkok 10700, Thailand

[5]Department of Biochemistry, Faculty of Science, Mahidol University, Bangkok 10400, Thailand

[6]Chulabhorn Research Institute, Kamphaeng Phet 6 Road, Laksi, Bangkok 10210, Thailand

[7]Center of Excellence on Environmental Health and Toxicology (EHT), OPS, Ministry of Higher Education, Science, Research and Innovation, Bangkok 10400, Thailand

***Corresponding author:** Tawatchai Thongkongkaew, Chemical Sciences Program, Chulabhorn Graduate Institute, Chulabhorn Royal Academy, Laksi, Bangkok 10210, Thailand.

Email: tawatchait@cgi.ac.th

Table of Contents

**Table S1.** Antioxidant, xanthine oxidase inhibition, and cytotoxicity of compounds **1-4** and **6-16**	4
**Figure S1.** ^{1}H NMR spectrum of 5′-hydroxyhericenes A-D (**1-4**) in $CDCl_3$ (300 MHz)	5
**Figure S2.** ^{13}C NMR spectrum of 5′-hydroxyhericenes A-D (**1-4**) in $CDCl_3$ (75 MHz)	5
**Figure S3.** DEPT-135 spectrum of 5′-hydroxyhericenes A-D (**1-4**) in $CDCl_3$ (300 MHz)	6
**Figure S4.** ^{1}H-^{1}H COSY spectrum of 5′-hydroxyhericenes A-D (**1-4**) in $CDCl_3$	6
**Figure S5.** HSQC spectrum of 5′-hydroxyhericenes A-D (**1-4**) in $CDCl_3$	7
**Figure S6.** HMBC spectrum of 5′-hydroxyhericenes A-D (**1-4**) in $CDCl_3$	7
**Figure S7.** NOESY spectrum of 5′-hydroxyhericenes A-D (**1-4**) in $CDCl_3$	8
**Figure S8.** ESI-HRMS of 5′-hydroxyhericenes A-D (**1-4**)	8
**Figure S9.** Extracted ion chromatogram of 5′-hydroxyhericene A (**1**)	9
**Figure S10.** Extracted ion chromatogram of 5′-hydroxyhericene B (**2**)	9
**Figure S11.** Extracted ion chromatogram of 5′-hydroxyhericene C (**3**)	10
**Figure S12.** Extracted ion chromatogram of 5′-hydroxyhericene D (**4**)	10
**Figure S13.** GCMS profiles of the hydrolysate from 5′-hydroxyhericenes A-D (**1-4**), 37 fatty acid methyl esters from standard mixture (37 FAM standard), and control	11
**Figure S14.** Confirmation the identity of fatty acid methyl ester by comparing with 37 FAM standard and NIST library	12
**Figure S15.** ESI-HRMS/MS of adenosine (**6**) isolated from *H. erinaceus*	14
**Figure S16.** ^{1}H NMR spectrum of commercially available adenosine and adenosine (**6**) isolated from *H. erinaceus* in DMSO-d6 (300 MHz)	14
**Figure S17.** ^{13}C NMR spectrum of commercially available adenosine and adenosine (**6**) isolated from *H. erinaceus* in DMSO-d6 (75 MHz)	15

Table S1. Antioxidant, xanthine oxidase inhibition, and cytotoxicity of compounds **1-4** and **6-16**

**Compound**	**Percent activity or IC_{50} (μM)**			**Percent cytotoxicity or IC_{50} (μM)**		
	**DPPH**	**IXO**	**XXO**	**T47D**	**MDA-MB-231**	**MRC-5**
**1-4**	4.0±0.0	(IC_{50} 7.3±0.6 μg mL^{-1})	ND	13.0±9.3	5.7±4.2	ND
**6**	11.5±0.7	27.0±7.9	IC_{50} 498.0±25.5	2.3±2.9	2.5±5.0	ND
**7 and 8**	5.5±0.7	32.0±3.6	16.0±4.2	6.0±3.2	0.0±0.0	ND
**9 and 10**	2.0±1.4	22.0±12.7	(IC_{50} 60.8±6.1 μg mL^{-1})	19.0±5.8	3.1±3.6	ND
**11**	8.0±1.4	28.7±2.9	41.5±0.7	28.3±4.9	17.2±3.8	ND
**12**	5.5±0.7	32.7±2.5	29.0±0.0	16.8±11.7	11.8±3.8	ND
**13**	13.5±2.1	43.3±5.0	42.5±9.2	IC_{50} 5.9±1.6	IC_{50} 57.5±5.4	IC_{50} 79.6±13.7
**14**	4.5±0.7	15.0±8.2	36.0±1.4	4.3±4.9	5.5±4.2	ND
**15**	2.0±0.0	15.0±9.6	29.0±1.4	8.8±2.8	6.2±4.1	ND
**16**	ND	ND	ND	39.0±0.0	25.9±8.6	ND
Ascorbic acid	IC_{50} 34.2±0.5					
Allopurinol		IC_{50} 4.1±0.1				
Gallic acid			IC_{50} 2.6±0.1			
Doxorubicin·HCl				IC_{50} 0.69±0.03	IC_{50} 2.21±0.23	IC_{50} 2.75±0.21

Note: Percent activity/percent cytotoxicity was determined at the cut-off concentration. When the percent activity/percent cytotoxicity is higher than 50, the IC_{50} value is reported. DPPH is a scavenging of diphenyl picrylhydracy radical. IXO is an inhibition of xanthine oxidase. XXO is an inhibition of reactive oxygen species generating by xanthine oxidase. T47D is a hormone-dependent breast cancer cell line. MDA-MB-231 is a hormone-independent breast cancer cell line. MRC-5 is a normal embryonic lung cell line. ND = not determined. The IC_{50} values of a mixture are reported in parentheses in μg mL^{-1}.

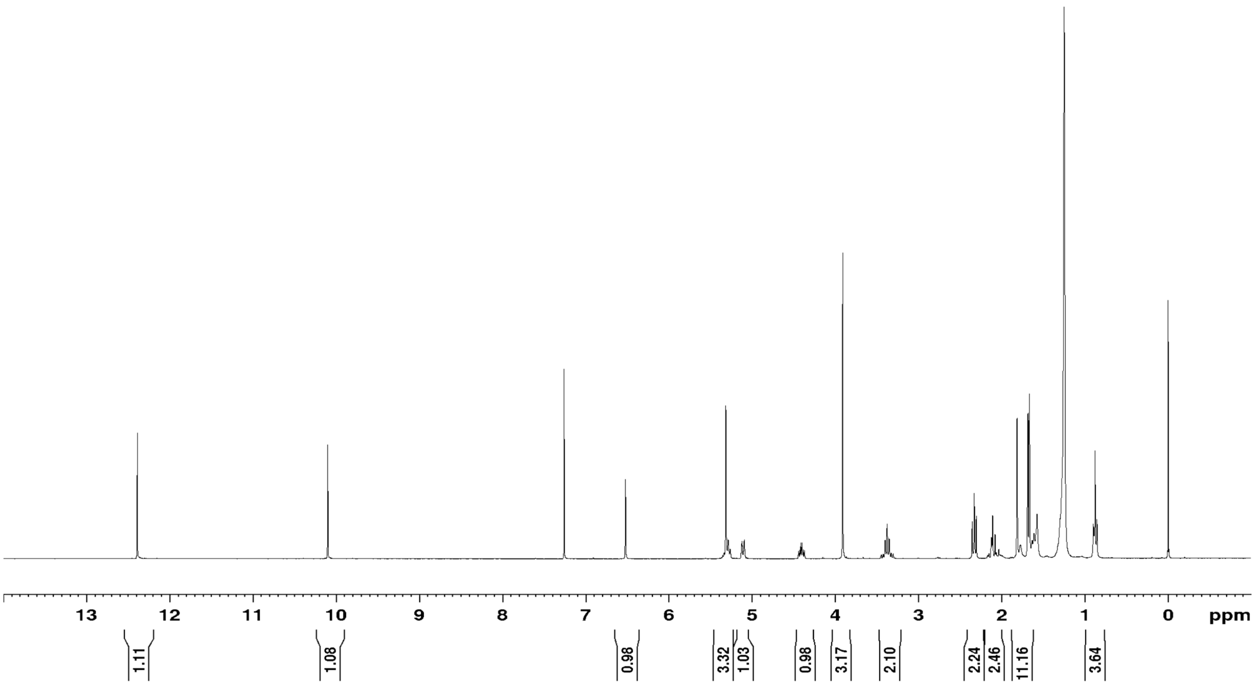

Figure S1. ^{1}H NMR spectrum of 5′-hydroxyhericenes A-D (**1**-**4**) in $CDCl_3$ (300 MHz)

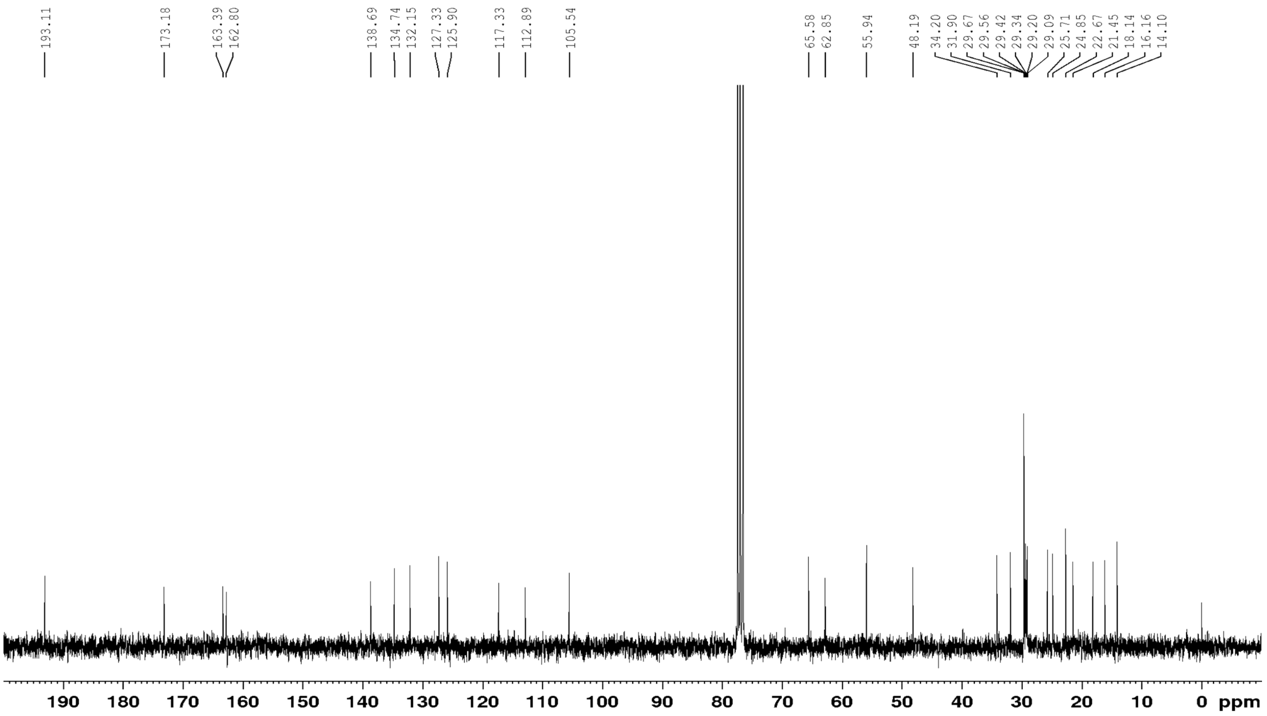

Figure S2. ^{13}C NMR spectrum of 5′-hydroxyhericenes A-D (**1**-**4**) in $CDCl_3$ (75 MHz)

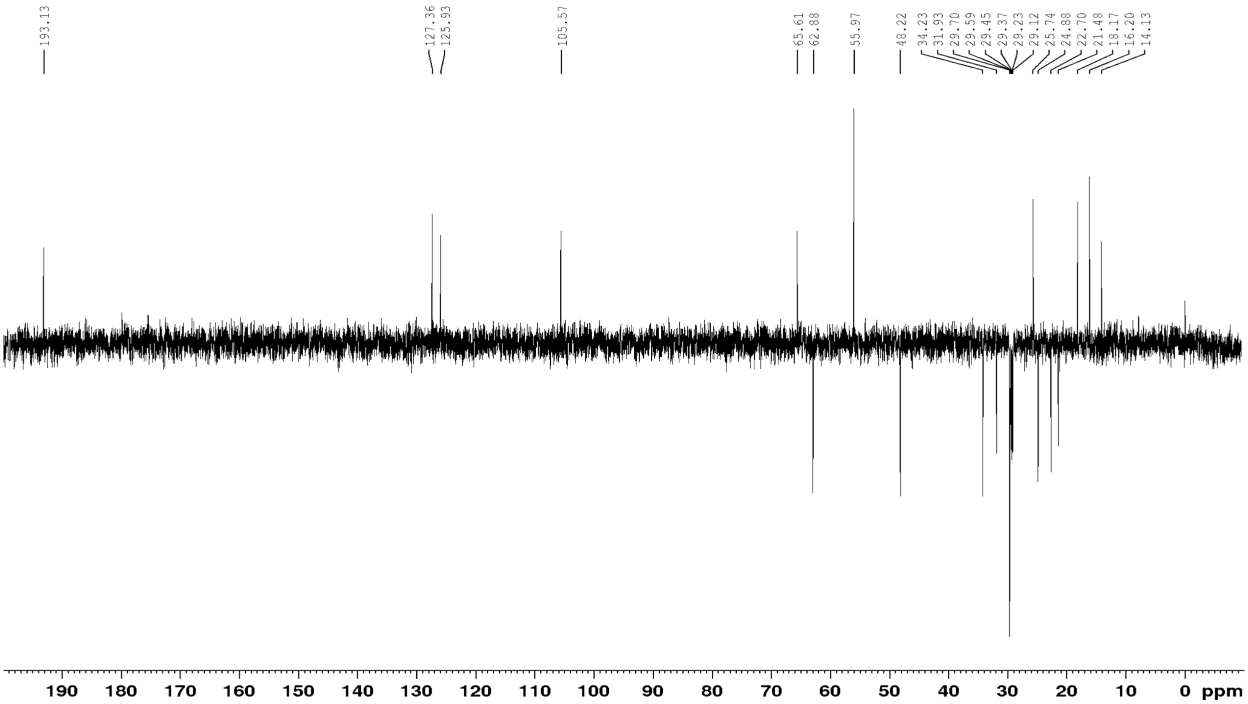

Figure S3. DEPT-135 spectrum of 5′-hydroxyhericenes A-D (**1-4**) in $CDCl_3$

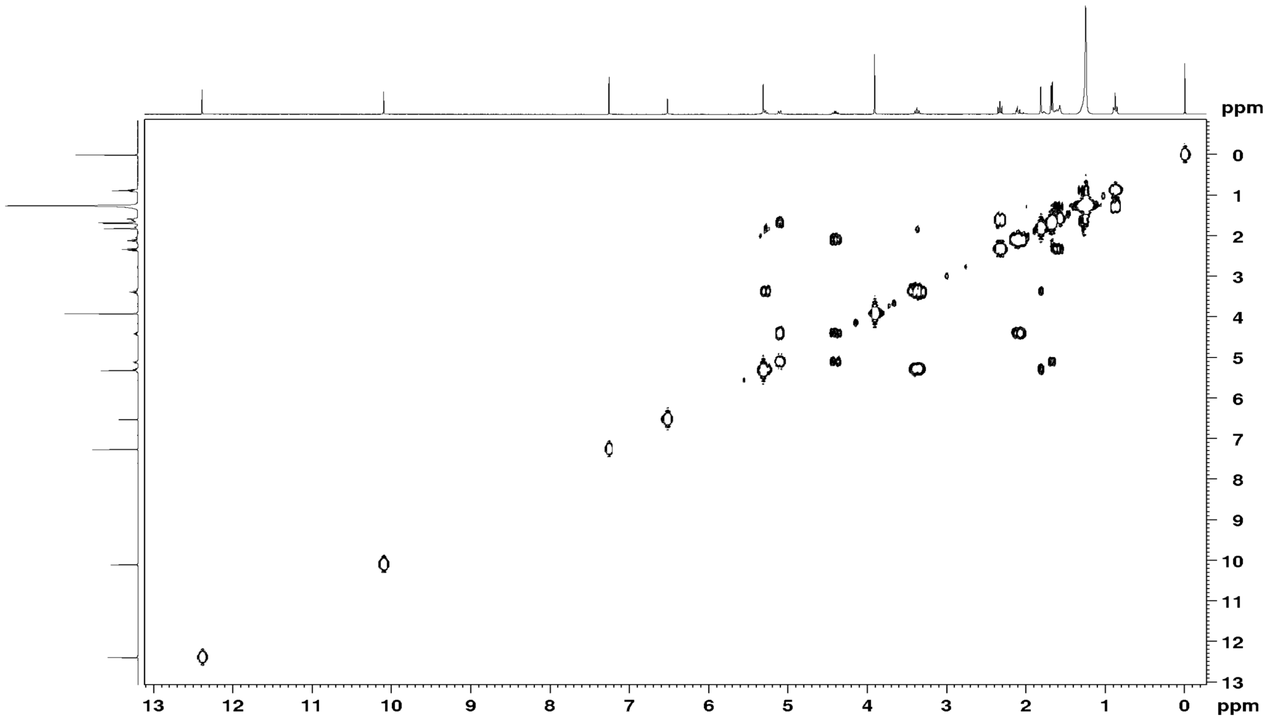

Figure S4. ¹H-¹H COSY spectrum of 5′-hydroxyhericenes A-D (**1-4**) in $CDCl_3$

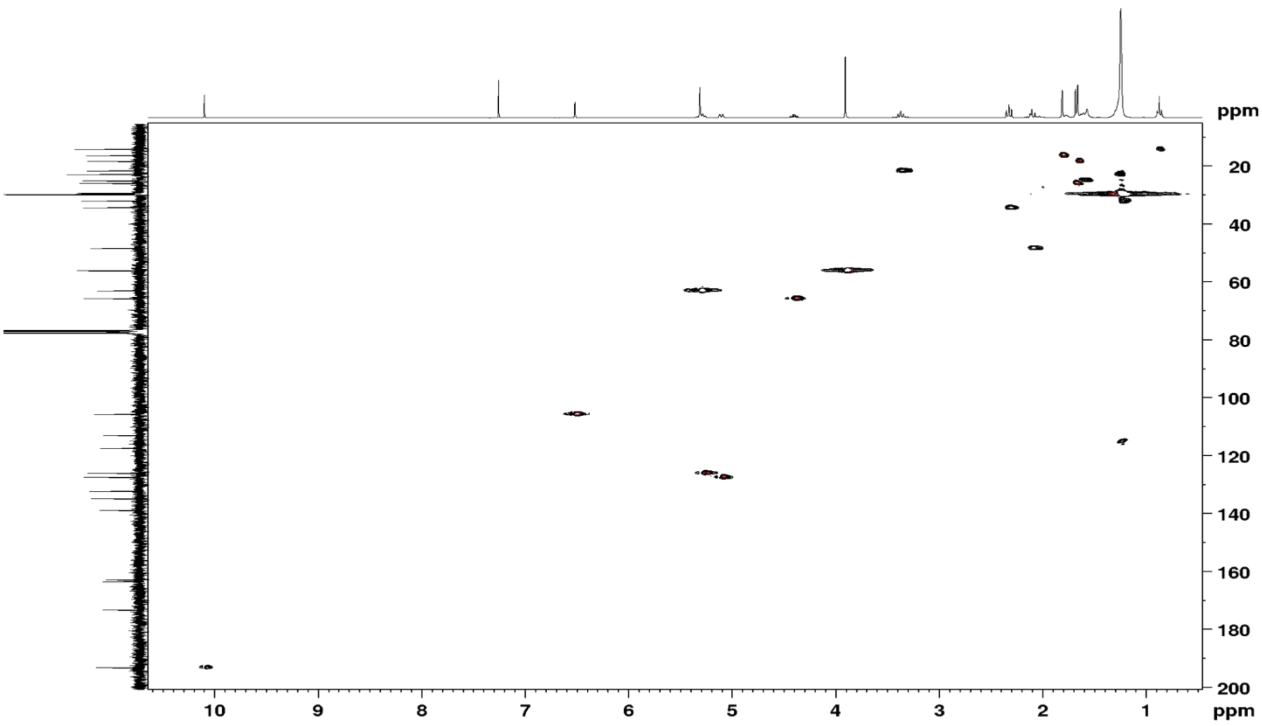

Figure S5. HSQC spectrum of 5´-hydroxyhericenes A-D (**1-4**) in $CDCl_3$

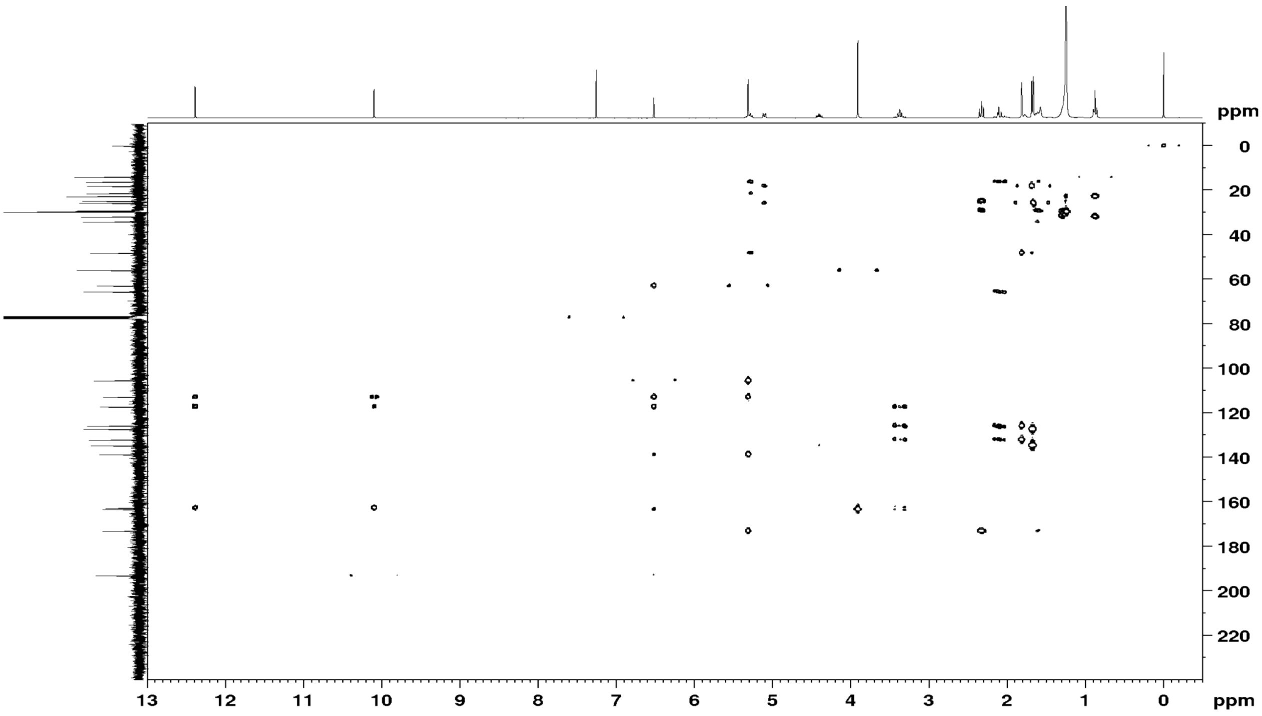

Figure S6. HMBC spectrum of 5´-hydroxyhericenes A-D (**1-4**) in $CDCl_3$

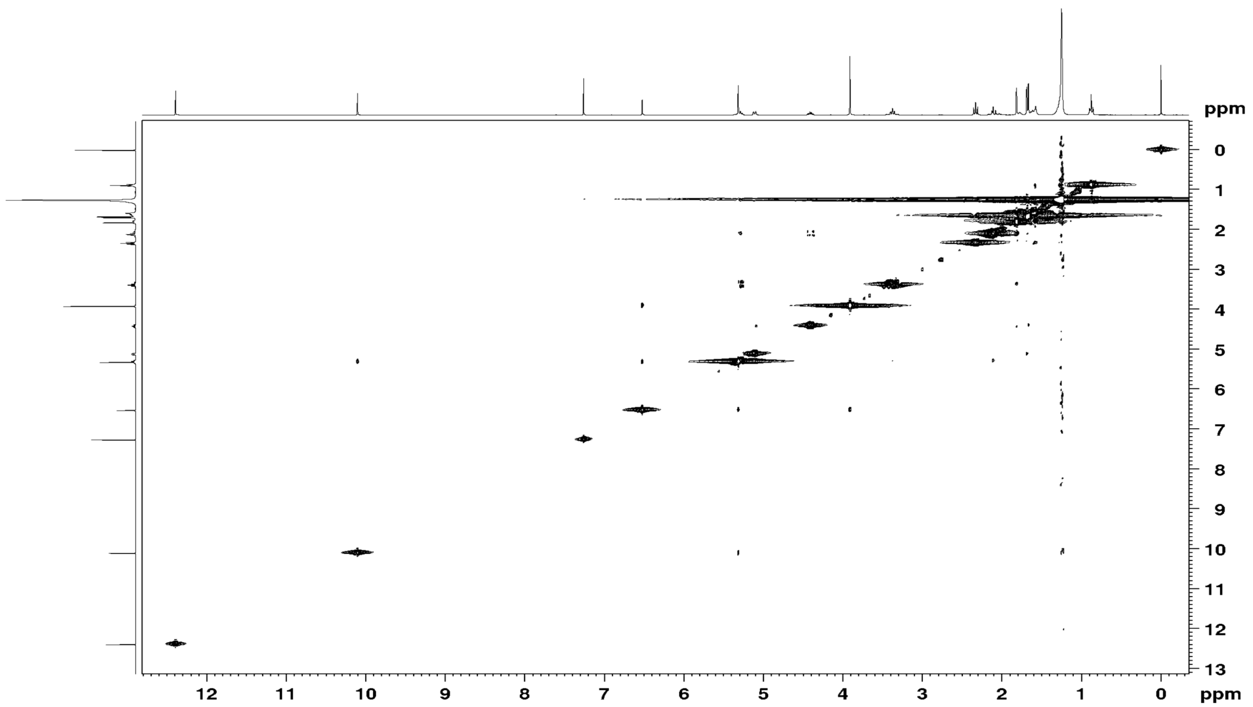

Figure S7. NOESY spectrum of 5´-hydroxyhericenes A-D (**1-4**) in $CDCl_3$

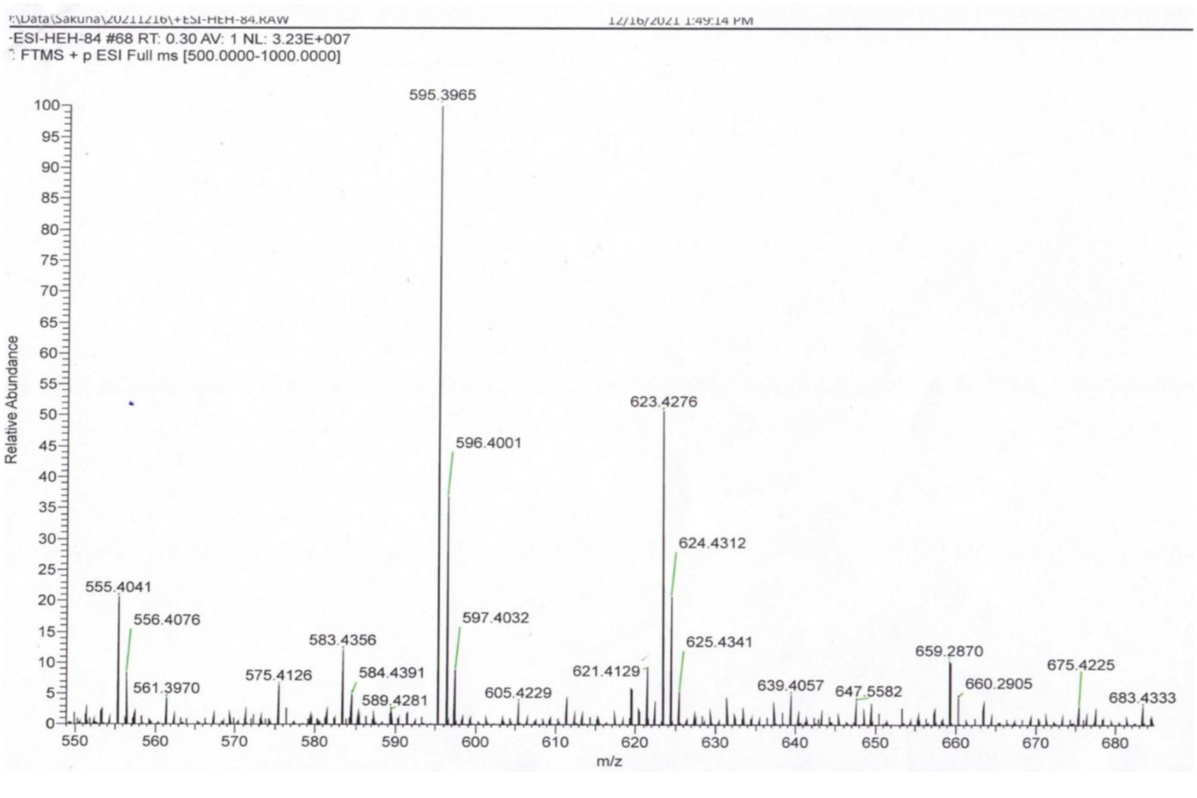

Figure S8. ESI-HRMS of 5´-hydroxyhericenes A-D (**1-4**)

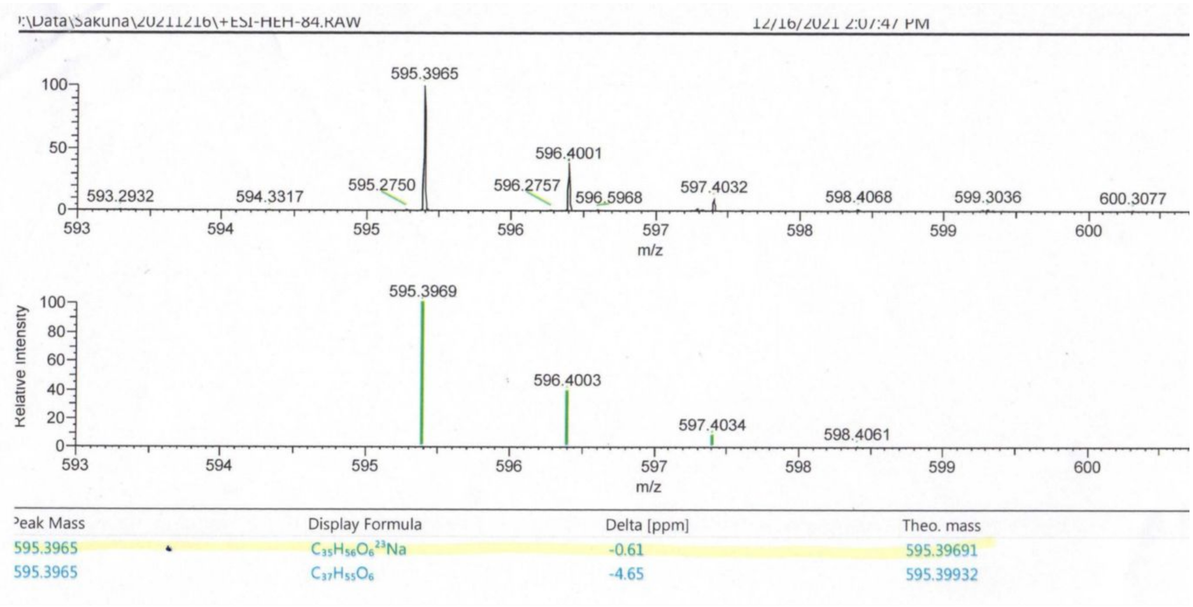

Figure S9. Extracted ion chromatogram of 5´-hydroxyhericene A (**1**)

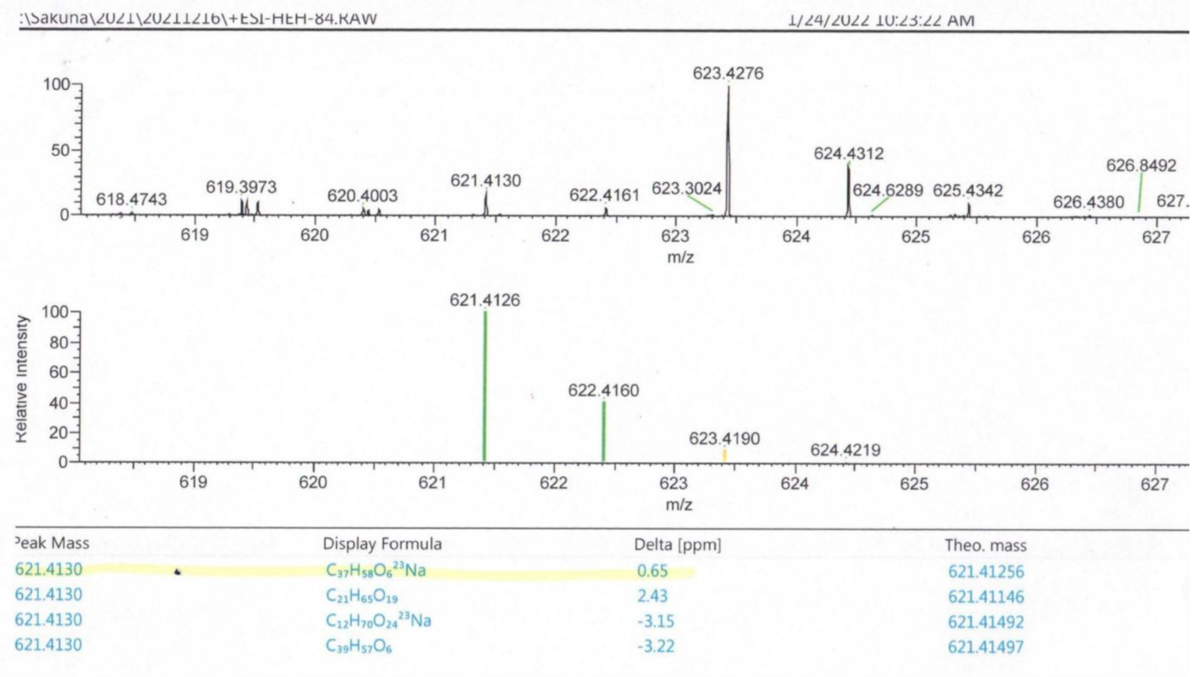

Figure S10. Extracted ion chromatogram of 5´-hydroxyhericene B (**2**)

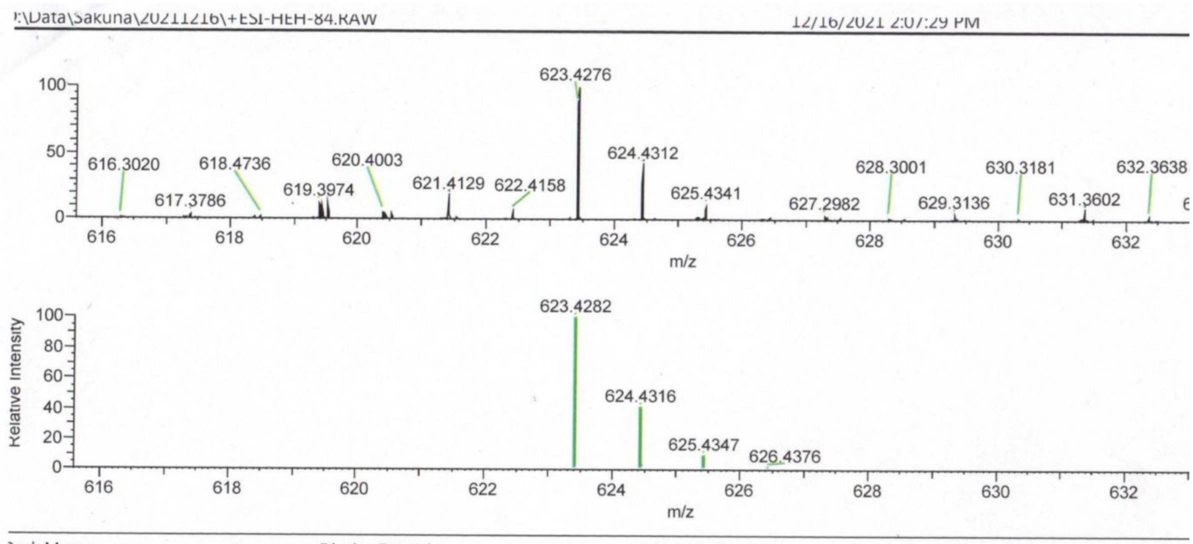

Peak Mass	Display Formula	Delta [ppm]	Theo. mass
623.4276	$C_{37}H_{60}O_6{}^{23}Na$	-0.96	623.42821
623.4276	$C_{46}H_{55}O$	4.60	623.42474
623.4276	$C_{39}H_{59}O_6$	-4.82	623.43062

Figure S11. Extracted ion chromatogram of 5´-hydroxyhericene C (**3**)

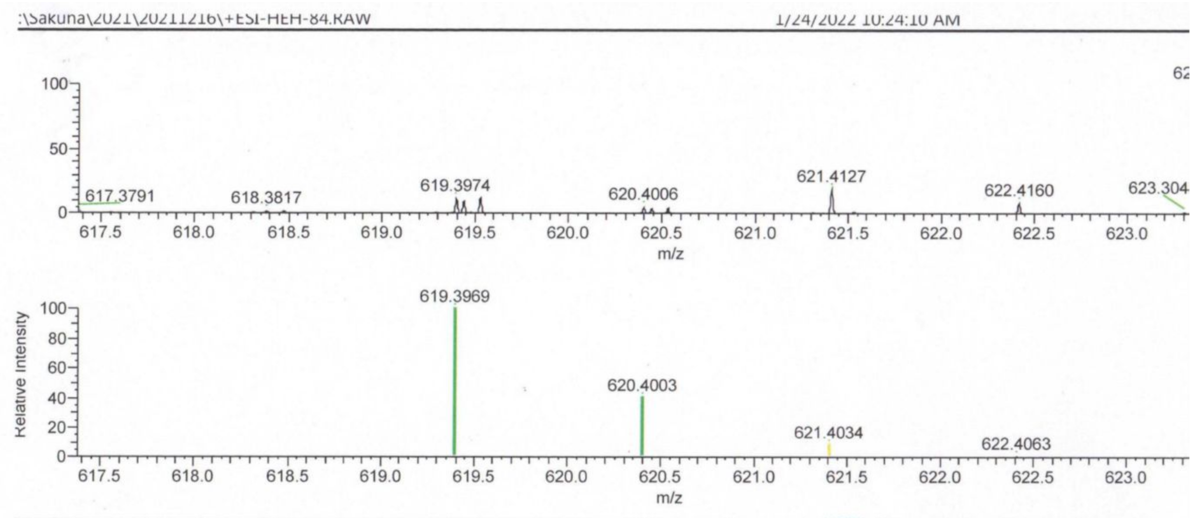

Peak Mass	Display Formula	Delta [ppm]	Theo. mass
619.3974	$C_{21}H_{63}O_{19}$	2.57	619.39581
619.3974	$C_{37}H_{56}O_6{}^{23}Na$	0.79	619.39691
619.3974	$C_{12}H_{68}O_{24}{}^{23}Na$	-3.03	619.39927
619.3974	$C_{39}H_{55}O_6$	-3.09	619.39932

Figure S12. Extracted ion chromatogram of 5´-hydroxyhericene D (**4**)

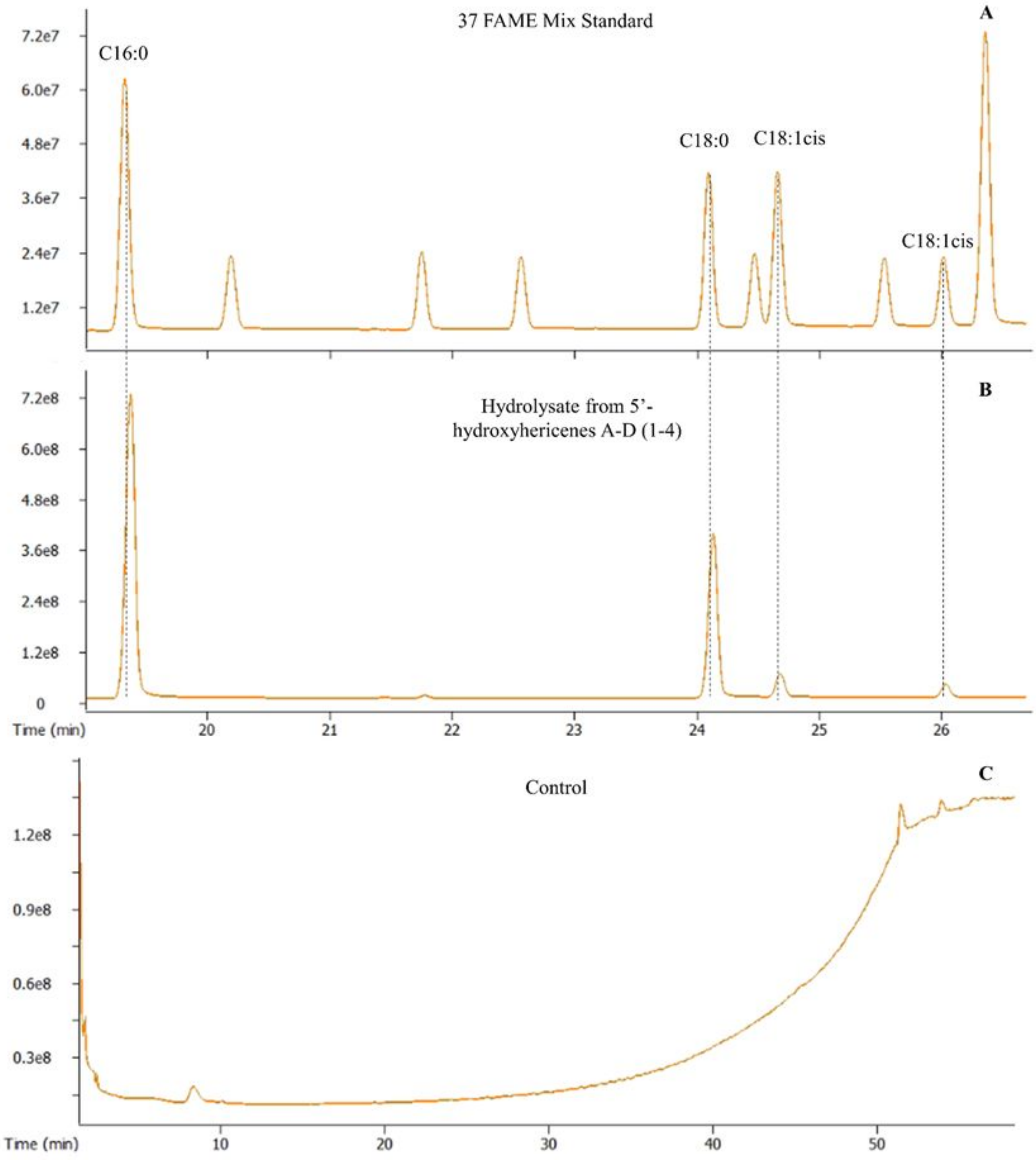

Figure S13. GCMS profiles of the hydrolysate from A) 37 fatty acid methyl ester mix standard (37 FAME standard), B) 5´-hydroxyhericenes A-D (**1**-**4**), and C) control

Methyl Palmitate

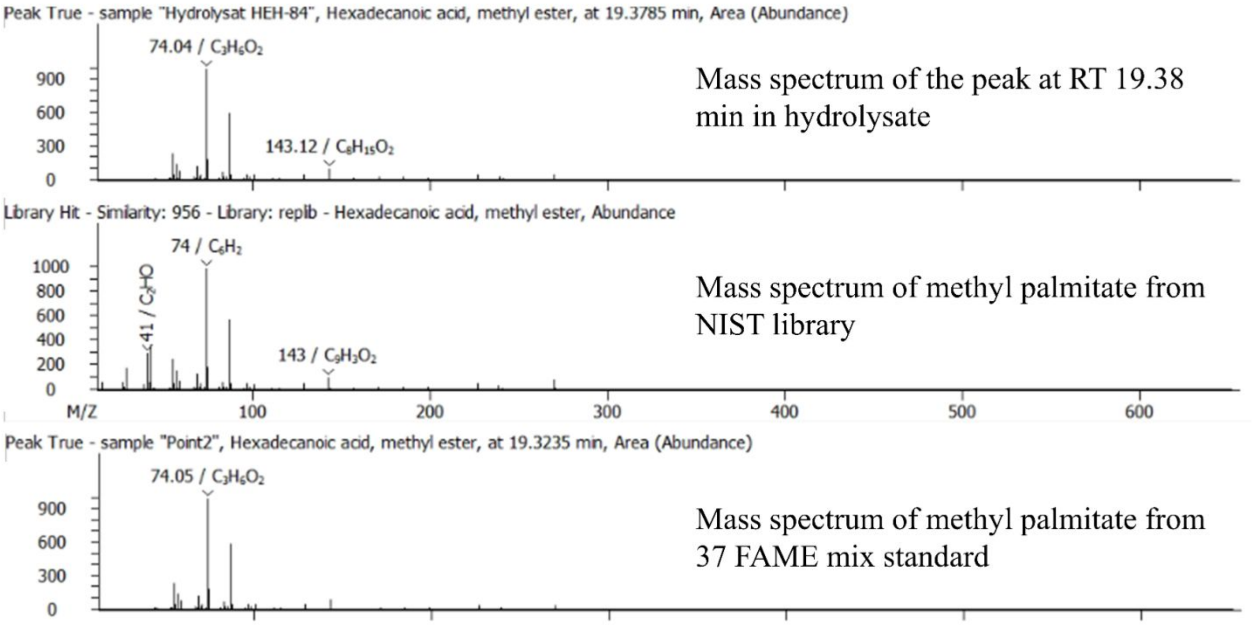

Methyl stearate

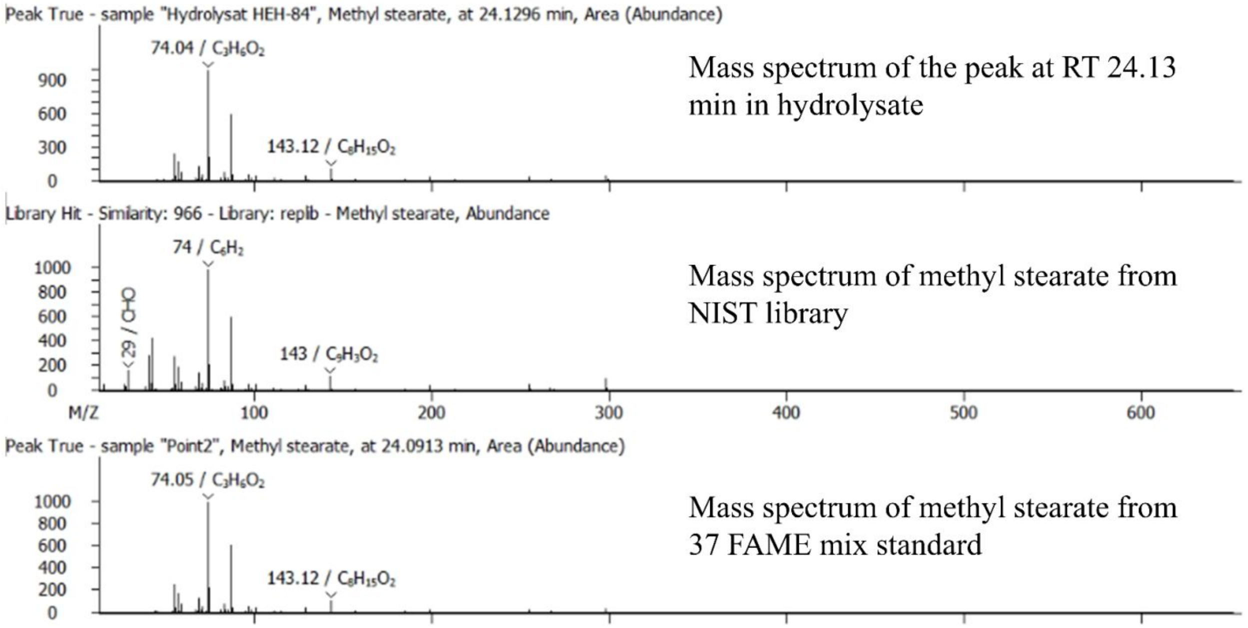

Methyl oleate (C18:1cis)

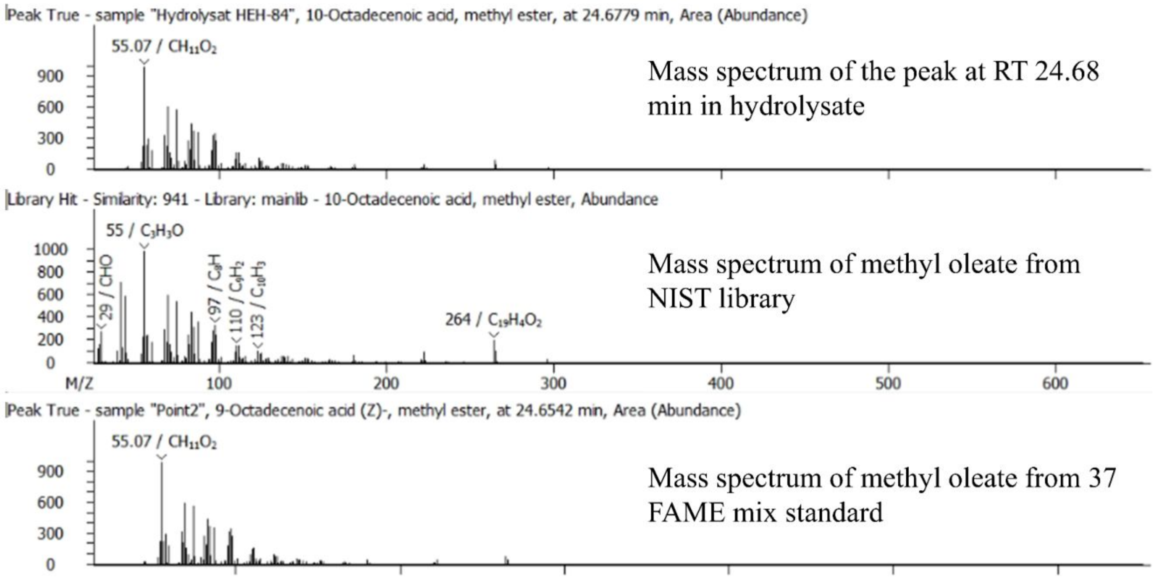

Methyl linoleate (C18:2cis)

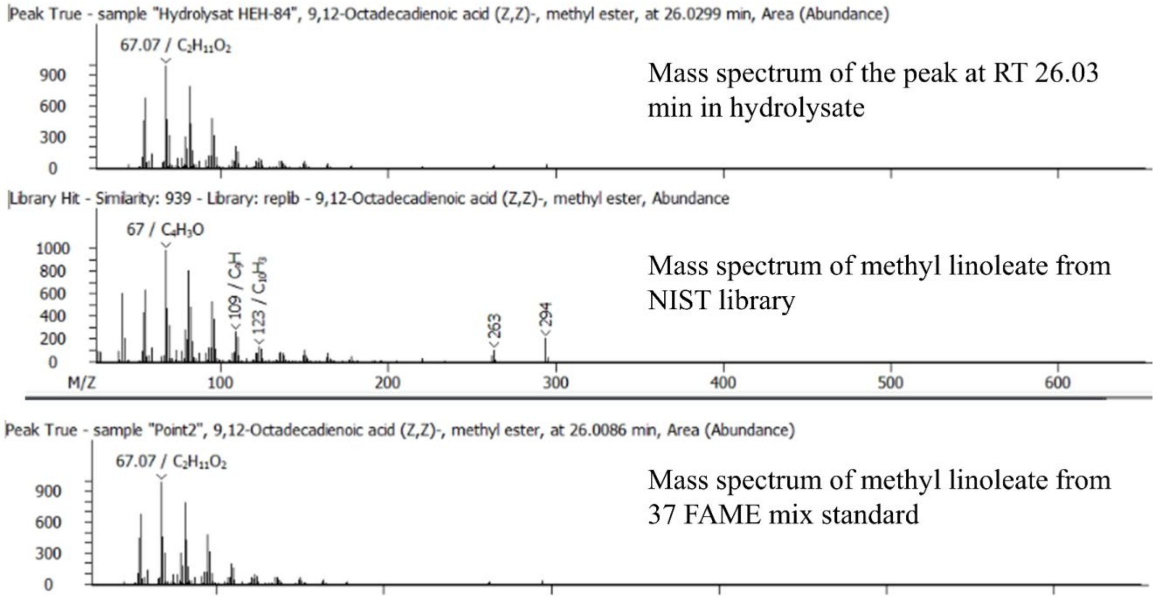

Figure S14. Identity confirmation of fatty acid methyl esters by comparing mass spectra of the peaks in hydrolysate with those obtained from the 37 FAME mix standard and NIST library.

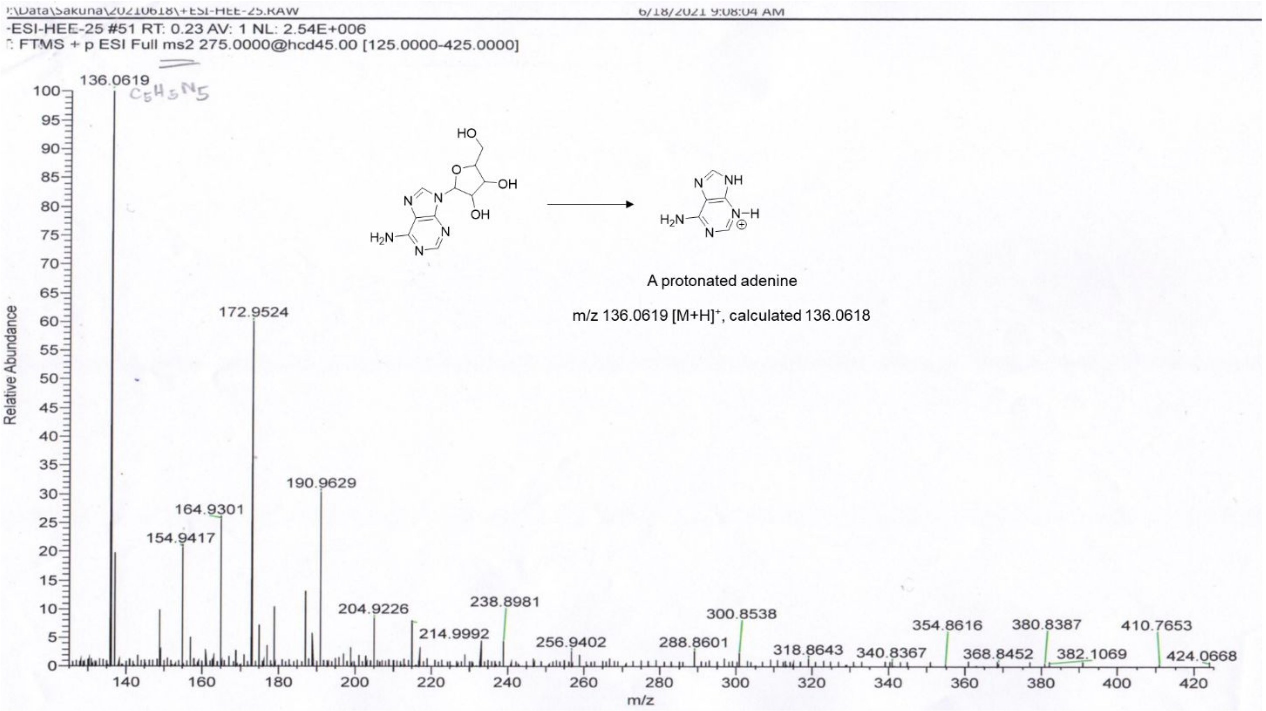

Figure S15. ESI-HRMS/MS of adenosine (**6**) isolated from *H. erinaceus*

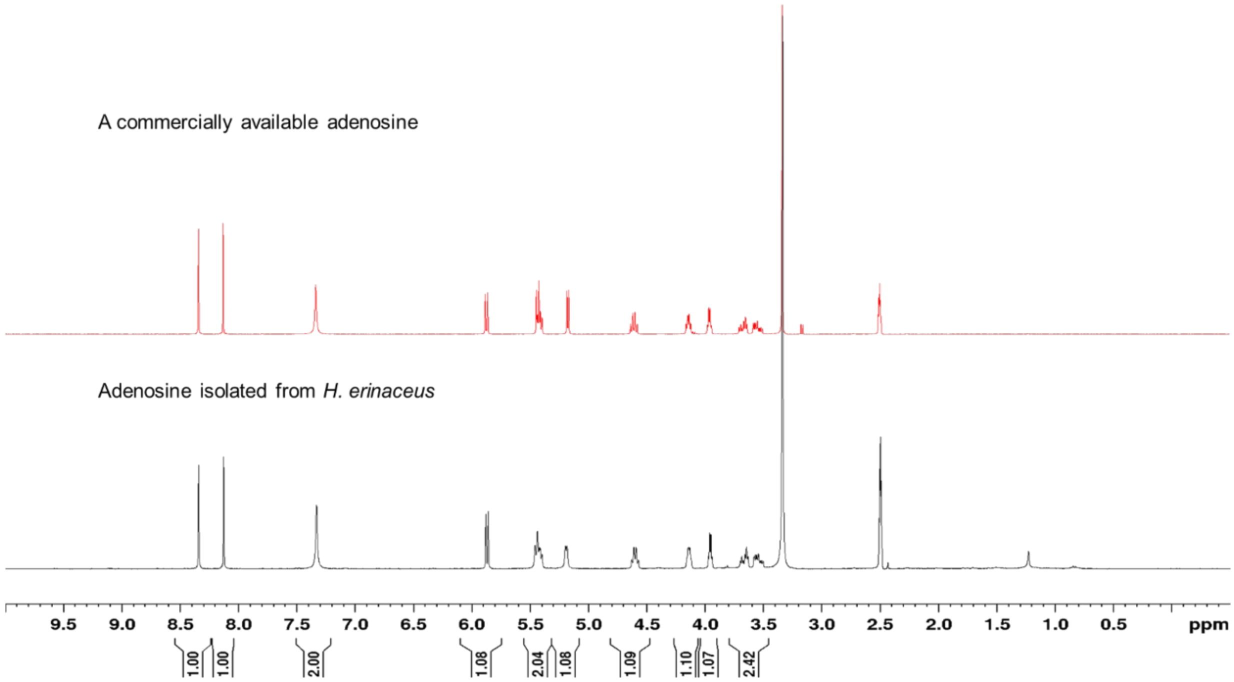

Figure S16. ^{1}H NMR spectrum of commercially available adenosine and adenosine (**6**) isolated *H. erinaceus* in DMSO-d6 (300 MHz)

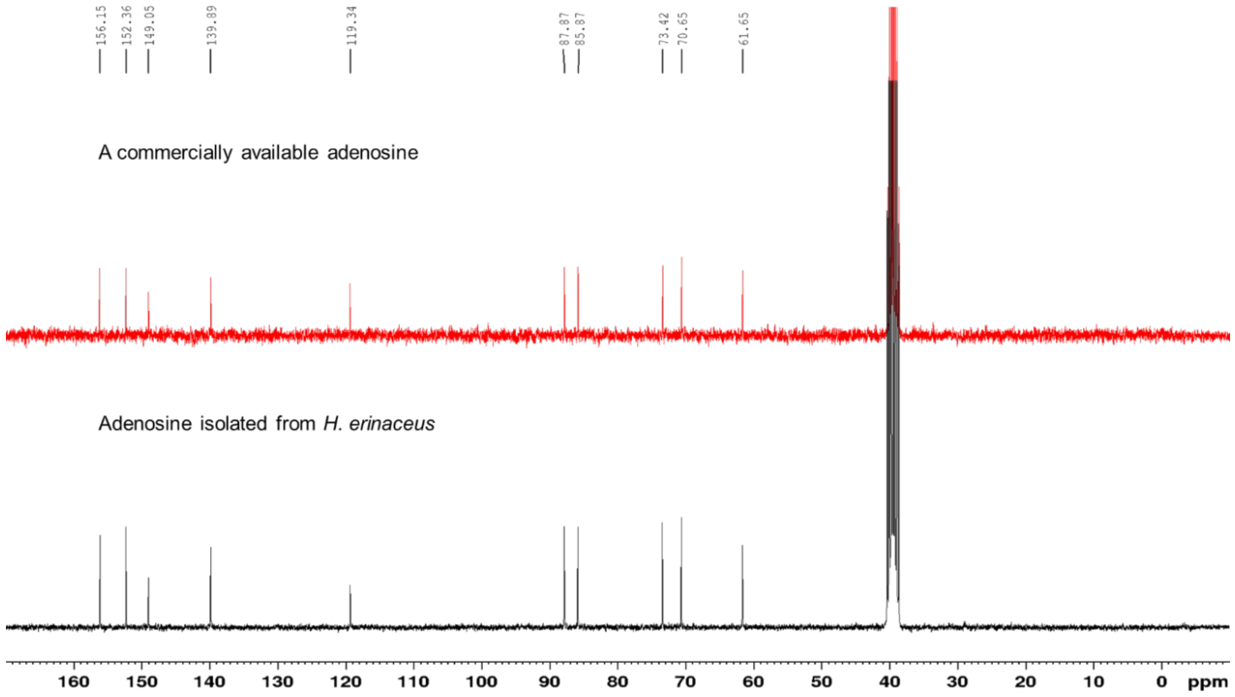

Figure S17. ^{13}C NMR spectrum of commercially available adenosine and adenosine (**6**) isolated from *H. erinaceus* in DMSO-d6 (75 MHz)
